# Associations of Interprofessional Adaptive Coordination and Intercultural Communication Competence with Missed Nursing Care in Remote Hospitals in South Papua, Indonesia

**DOI:** 10.3390/healthcare14203470

**Published:** 2026-10-15

**Authors:** Natalin Allorerung, Ferdi Antonio, Andri Hondir

**Affiliations:** Department of Hospital Administration, Pelita Harapan University, Jakarta 12930, Indonesia; natalinallorerung@gmail.com (N.A.); andrihondir@yahoo.com (A.H.)

**Keywords:** missed nursing care, patient safety, adaptive teamwork, interprofessional coordination, intercultural communication, nursing workforce, remote hospitals, healthcare quality

## Abstract

**Highlights:**

**What are the main findings?**

• Risk pattern recognition and safety sensemaking were positively associated with interprofessional adaptive coordination, which was negatively associated with perceived missed nursing care. Both capabilities also showed significant indirect associations with missed nursing care through interprofessional adaptive coordination.
• Intercultural communication competence significantly moderated the association between interprofessional adaptive coordination and missed nursing care, with a stronger negative association at higher levels of intercultural communication competence.

**What are the implications of the main findings?**

• Safety-related cognitive capabilities and interprofessional adaptive coordination warrant consideration in patient safety improvement strategies for remote hospitals.
• Culturally responsive communication may be an important consideration when developing interprofessional coordination initiatives to address missed nursing care in multicultural healthcare settings.

**Abstract:**

**Background:** Missed nursing care remains an important patient safety concern in geographically isolated and culturally diverse healthcare settings. Evidence remains limited on how cognitive safety capabilities, interprofessional coordination, and intercultural communication are associated with missed nursing care in remote multicultural settings. **Methods:** A cross-sectional survey was conducted among 312 nurses from four hospitals in Merauke Regency, South Papua, Indonesia. Data were analyzed using Partial Least Squares Structural Equation Modeling (PLS-SEM). **Results:** Risk pattern recognition, safety sensemaking, and learning from events orientation were positively associated with nurse moral resilience. Risk pattern recognition, safety sensemaking, and nurse moral resilience were also positively associated with interprofessional adaptive coordination, whereas learning from events orientation was not statistically associated with it. Interprofessional adaptive coordination was negatively associated with perceived missed nursing care, while nurse moral resilience showed no significant direct association. Nurse moral resilience also showed a significant indirect association with missed nursing care through interprofessional adaptive coordination. Intercultural communication competence moderated the association between interprofessional adaptive coordination and perceived missed nursing care, with a more negative association at higher levels of intercultural communication competence. **Conclusions:** The findings position interprofessional adaptive coordination as a distinctive relational component of the pattern surrounding perceived missed nursing care, with intercultural communication competence representing a pathway-specific contextual condition. The study provides an explanatory perspective on missed nursing care in a remote, culturally diverse healthcare setting.

## 1. Introduction

Patient safety remains a major challenge for healthcare systems worldwide, particularly within nursing services, where the continuity and quality of patient care depend on adequate staffing, effective teamwork, and organizational support [1,2]. One of the most widely recognized indicators of compromised patient safety is missed nursing care (MNC), defined as any required nursing care that is delayed, partially completed, or omitted during patient care delivery [3]. As originally conceptualized, missed nursing care represents an error of omission, reflecting necessary nursing activities that patients should have received but did not receive [3,4]. Previous studies have consistently reported that higher levels of missed nursing care are associated with adverse patient outcomes, including medication errors, preventable complications, healthcare-associated infections, prolonged hospitalization, lower patient satisfaction, and increased mortality risk, underscoring its importance as an indicator of healthcare quality and patient safety [4,5,6,7].

Although missed nursing care has been widely investigated in hospital settings, most empirical evidence has been generated from urban or relatively well-resourced healthcare systems [5,6]. Comparatively less is known about missed nursing care in geographically isolated hospitals, where healthcare professionals frequently work under conditions characterized by shortages of skilled personnel, limited access to specialist services, logistical constraints, and restricted opportunities for continuing professional development [8,9]. These conditions may create additional challenges for frontline nurses in maintaining continuity of care. South Papua, Indonesia, represents one such setting, where hospitals serve dispersed populations across remote areas while operating under substantial workforce and infrastructure challenges [9]. Examining missed nursing care within this context may therefore contribute evidence from a healthcare environment that remains underrepresented in the current patient safety literature.

In addition to geographical isolation, healthcare delivery in South Papua occurs within a highly multicultural environment. The province is characterized by substantial ethnic, linguistic, and cultural diversity, where Indigenous Papuan communities coexist with healthcare professionals and migrant populations from different regions of Indonesia [10,11]. This diversity is relevant to healthcare delivery because differences in communication styles, health beliefs, expectations regarding illness and treatment, and interactions between healthcare providers and patients may introduce additional complexity into routine care [10,11,12,13]. Previous studies have also suggested that local cultural values and social norms are relevant to professional behaviours, decision-making processes, and workplace interactions within Indonesian healthcare settings [10,11,12,13]. These contextual characteristics underscore the relevance of culturally responsive communication when examining patient care in remote and multicultural hospital settings.

The importance of intercultural communication has received increasing attention in healthcare, particularly in relation to culturally responsive communication between healthcare professionals and patients [13,14,15]. Such communication is relevant to healthcare delivery in contexts characterized by differences in language, cultural values, health beliefs, and expectations regarding care. However, previous research has primarily examined intercultural competence in relation to communication between healthcare professionals and patients or within educational settings, with comparatively less attention to its relevance in remote hospital environments. In such settings, nurses provide care within conditions of operational uncertainty and cultural diversity, making intercultural communication competence a relevant contextual capability when examining factors associated with missed nursing care [13,14,15]. Importantly, in the present study, intercultural communication competence refers primarily to nurses’ culturally responsive communication with patients rather than communication among healthcare professionals. This distinction is important because interprofessional communication and coordination are represented separately in the model through interprofessional adaptive coordination.

Beyond communication challenges, healthcare professionals working in remote hospitals also encounter clinical uncertainty, workforce shortages, and ethical pressures that are relevant to the continuity of patient care. These conditions highlight the relevance of adaptive capabilities, including the capacity to recognize emerging risks, interpret complex situations, and coordinate responses under changing clinical conditions [16,17,18,19,20,21]. Although these capabilities have been examined across different streams of healthcare research, their relationships with missed nursing care have received comparatively limited attention when considered together, particularly in geographically isolated and culturally diverse hospital settings.

This area of inquiry is particularly relevant in multicultural healthcare environments, where communication barriers, cultural differences, varying expectations regarding professional roles, and differences in health beliefs may be relevant to collaboration and clinical decision-making. Although intercultural communication competence has received increasing attention in healthcare, its potential role in the relationship between adaptive nursing capabilities and missed nursing care remains comparatively underexamined in multicultural and resource-constrained hospital settings [13,14,15,16,17]. Examining these relationships may provide further insight into how individual and collaborative capabilities are associated with continuity of nursing care under conditions of cultural and operational complexity.

Recent studies have examined collaborative processes as an important dimension of safe nursing care, particularly in healthcare environments characterized by uncertainty and changing clinical demands. Interprofessional coordination involves timely information exchange, shared decision-making, and responses to emerging patient needs, and has therefore been examined in relation to continuity of care [8,9,18,20,21,22,23]. At the individual level, nurse moral resilience has been associated with the capacity to navigate ethically challenging situations, maintain professional integrity, and continue providing patient care under demanding working conditions [20,21,24,25]. Taken together, these strands of research indicate that both individual and collaborative capabilities are relevant perspectives for examining missed nursing care alongside structural and organizational conditions.

Although these areas have developed substantially, the relevant evidence remains distributed across several streams of research. Research on missed nursing care has largely focused on structural and work-related factors, such as staffing adequacy, workload, and characteristics of the work environment, whereas research on cognitive safety capabilities, moral resilience, adaptive interprofessional coordination, and intercultural communication has generally developed along separate lines [5,6,7,8,13,16,17,18,19,20]. This separation leaves limited empirical understanding of how these individual and collaborative capabilities are associated with missed nursing care when considered together, particularly in geographically isolated and culturally diverse hospital settings characterized by clinical uncertainty and cultural complexity.

Existing research has generally examined resilience, teamwork, organizational learning, communication, and patient safety as related but distinct areas of inquiry [5,6,13,16,17,20,22]. A theoretical perspective that connects these areas may therefore help explain how nurses interpret clinical uncertainty, develop shared understanding, and coordinate responses in geographically isolated and culturally diverse hospital settings. In this context, Sensemaking Theory provides a relevant perspective because it emphasizes how individuals interpret clinical cues, construct meaning, and adapt their actions when confronted with uncertain and evolving situations.

Against this background, the present study draws on Weick’s Sensemaking Theory as its primary theoretical perspective [26,27]. In healthcare settings, sensemaking refers to how professionals interpret clinical information and events, identify relevant cues, and develop an understanding of situations that may be uncertain or changing. This perspective is particularly relevant to remote hospital settings, where nurses may encounter complex clinical situations while working within geographically and operationally constrained environments [28]. Within this framework, the present study considers three cognitive safety capabilities, risk pattern recognition, safety sensemaking, and learning from events orientation, as related aspects of how healthcare professionals recognize and interpret clinical situations and patient safety information. These capabilities are examined alongside nurse moral resilience and interprofessional adaptive coordination as adaptive capabilities, while intercultural communication competence is considered as a contextual capability within the multicultural healthcare environment.

Building on this theoretical perspective, the proposed framework considers nurse moral resilience and interprofessional adaptive coordination as adaptive capabilities examined in relation to missed nursing care alongside the cognitive safety capabilities described above. Nurse moral resilience refers to healthcare professionals’ capacity to maintain professional integrity when facing ethical and clinical challenges [20,21,24,25]. Interprofessional adaptive coordination refers to how healthcare professionals exchange information, adjust roles, and coordinate actions in response to changing clinical situations [8,22,23]. Given the multicultural characteristics of healthcare delivery in South Papua, intercultural communication competence is further considered as a contextual capability, particularly in relation to culturally responsive communication with patients. In the present framework, intercultural communication competence is examined as a moderator of the association between interprofessional adaptive coordination and missed nursing care [13,14,15].

The present study therefore develops and examines an integrated conceptual framework that brings together cognitive safety capabilities, nurse moral resilience, interprofessional adaptive coordination, and intercultural communication competence within a Sensemaking Theory-informed framework. The study focuses on frontline nurses working in remote hospitals in South Papua, Indonesia, a healthcare setting characterized by geographical isolation and substantial cultural diversity. Using a cross-sectional survey design, the study examines the associations among these constructs and evaluates whether intercultural communication competence moderates the association between interprofessional adaptive coordination and missed nursing care. By providing empirical evidence from this underrepresented healthcare setting, the study contributes to a more context-sensitive understanding of individual, collaborative, and intercultural capabilities associated with missed nursing care in resource-constrained and multicultural hospital environments.

## 2. Methods

### 2.1. Study Design

This study employed a cross-sectional quantitative survey design to examine the hypothesized associations among cognitive safety capabilities, nurse moral resilience, interprofessional adaptive coordination, intercultural communication competence, and missed nursing care in remote hospital settings. A cross-sectional design was appropriate because all study variables were measured at a single point in time, allowing the proposed associations among the constructs to be examined without implying causal inference. The proposed research model was evaluated using Partial Least Squares Structural Equation Modeling (PLS-SEM), which is suitable for examining complex models involving multiple latent constructs, including mediation and moderation, with an emphasis on prediction and theory development [29].

### 2.2. Study Setting and Participants

The study was conducted in four general hospitals located in Merauke Regency, South Papua Province, Indonesia, a geographically remote region characterized by dispersed populations, workforce shortages, and limited access to specialist healthcare services. These hospitals provide secondary healthcare services for urban, rural, and remote communities across the southern part of Papua, where nurses deliver care under conditions of resource limitations and considerable operational complexity. This setting was selected because it represents a geographically remote and culturally diverse healthcare environment that remains underrepresented in empirical research on missed nursing care. The final sample comprised 312 nurses recruited from four participating hospitals in Merauke Regency, South Papua: RSUD Merauke (*n* = 117), RS Bunda Pengharapan (*n* = 98), RS Tentara Angkatan Darat Merauke (*n* = 51), and RS Angkatan Laut Merauke (*n* = 46).

A non-probability sampling approach was used, with all nurses who met the predefined eligibility criteria across the four participating hospitals invited to participate. This approach was aligned with the study objective of examining associations among the study constructs within the participating hospital settings and allowed eligible nurses across the participating clinical units to be invited without selecting a subset through probability-based sampling. Eligible participants were registered nurses providing direct patient care in inpatient wards, emergency departments, intensive care units, or other clinical service units, with at least six months of clinical experience. Nurses working exclusively in administrative positions without routine patient care responsibilities or those on extended leave during the data collection period were excluded. Based on these criteria, 338 nurses were eligible for participation. Of these, 320 returned questionnaires, yielding a response rate of 94.7%. After excluding eight questionnaires due to incomplete responses, 312 valid questionnaires were retained for the final analysis.

The minimum required sample size was estimated using G*Power 3.1 based on a power analysis [29], assuming six predictors, a medium effect size (f^2^ = 0.15), a significance level of 0.05, and statistical power of 0.90. The calculation indicated a minimum sample size of 123 participants. This value was treated as the minimum analytical requirement, while recruitment was not restricted to this number. Consistent with the non-probability sampling approach, all nurses who met the predefined eligibility criteria across the four participating hospitals were invited to participate. This approach allowed the study to include eligible nurses across the participating clinical units and maximize the number of observations available within the study setting. The final analytical sample comprised 312 nurses, exceeding the minimum sample size indicated by the power analysis [29].

### 2.3. Questionnaire Development and Data Collection

The study questionnaire was developed by adapting previously validated instruments that measured each construct included in the proposed conceptual framework. All constructs were measured using a five-point Likert scale ranging from 1 (strongly disagree) to 5 (strongly agree). The measurement instruments were developed through a sequential process of translation, contextual adaptation, expert evaluation, and pilot testing. The original measurement items were first translated into Indonesian by a bilingual expert with experience in healthcare terminology. The translated items were then reviewed and contextually modified to ensure linguistic clarity, conceptual equivalence, and relevance to Indonesian nurses and their clinical practice. Given the contextual adaptation of the instruments, equivalence of meaning was further assessed through multidisciplinary expert review and subsequent pilot testing.

For established constructs, the substantive meaning of the original items was retained while wording was adapted to reflect the terminology, responsibilities, and experiences of nurses in the study setting. The measurement sources comprised Yang et al. (2024) for missed nursing care (MNC) [6] and O’Keeffe et al. (2015) [30]; Shyu et al. (2017) and Rashvand et al. (2016) for risk pattern recognition (RPR) [31,32]; O’Keeffe et al. (2015), Groves et al. (2021), Vogus and Sutcliffe (2007) for safety sensemaking (SSM) [30,33,34]; Bahmanpour et al. (2021), Öztürk and Kahriman (2020), and Ginsburg et al. (2009) for learning from events orientation (LEO) [35,36,37]; Heinze et al. (2021) and Tian et al. (2023) for nurse moral resilience (NRM) [38,39]; Hu et al. (2026) and Kenaszchuk et al. (2010) for interprofessional adaptive coordination (IPC) [40,41]; and Bernhard et al. (2015), Tsatzali et al. (2025), and Sakamoto et al. (2025) for intercultural communication competence (ITCC) [42,43,44].

For the missed nursing care (MNC) construct, six candidate indicators were developed through contextual adaptation of three established sources: the Missed Intensive Nursing Care Scale (MINCS) developed by Yang et al. (2024) [6], the original MISSCARE Survey developed by Kalisch and Williams (2009) [45], and the revised MISSCARE Survey reported by Dabney et al. (2019) [46]. Rather than adopting items verbatim from a single instrument, each candidate indicator was mapped individually across the three sources according to its conceptual and domain-level correspondence with missed nursing care. The candidate items were subsequently translated and contextually adapted to reflect the terminology, responsibilities, and clinical circumstances of nurses working in the participating remote hospitals while preserving the substantive meaning of the source content. The adapted items were then subjected to expert review for conceptual relevance, clarity, and contextual appropriateness before empirical measurement assessment.

MNC was operationalized as nurses’ perceived occurrence of delayed, incomplete, or omitted nursing care activities within routine clinical practice. Responses were recorded on a five-point agreement scale ranging from 1 (strongly disagree) to 5 (strongly agree), with higher scores indicating stronger agreement that the described missed-care condition had occurred and, therefore, a higher perceived occurrence of MNC. No reverse coding was applied. Accordingly, the reported MNC item means represent the degree of nurses’ agreement with the occurrence of missed nursing care rather than a direct frequency count of missed-care events. Following contextual adaptation, the indicators were specified as reflective measures, treating the observed items as manifestations of an underlying perceived missed-care construct [29]. Although the candidate indicators represented different forms and circumstances of missed care, they were initially considered observable manifestations of the broader MNC construct as conceptualized in this study. The final indicator set was determined through the subsequent measurement assessment rather than through source adoption alone. Thus, the resulting measure represents a context-adapted assessment of nurses’ perceived missed nursing care and should not be interpreted as a frequency-based replication of the established MISSCARE instruments [6,45,46].

Following translation and contextual adaptation, all candidate items across the study constructs were subjected to face and content validity assessment by a multidisciplinary expert panel consisting of two academic experts from Pelita Harapan University, one senior nurse, one hospital chief nurse, and one field surveyor. The panel assessed the clarity, comprehensibility, conceptual relevance, and contextual appropriateness of each item for nursing practice. Minor wording refinements were made where necessary while preserving the intended meaning of the source measures. Content validity was subsequently assessed using the Content Validity Index (CVI), with the S-CVI/Ave values across all study constructs ranging from 0.750 to 0.850. The CVI results were interpreted together with the qualitative feedback from the expert panel, consistent with recommendations for evaluating content validity at both the item and scale levels (Polit et al., 2007) [47]. The complete Indonesian questionnaire was subsequently pilot-tested with 30 nurses who were not included in the main study sample. The pilot test assessed item comprehensibility, response appropriateness, and preliminary internal consistency before the main data collection. Cronbach’s alpha coefficients for all constructs were above 0.70, indicating satisfactory preliminary internal consistency of the adapted questionnaire. The pilot findings therefore supported the clarity and preliminary reliability of the measurement instruments before their administration in the main study.

Data were collected from 16 April to 31 May 2026 using a structured self-administered questionnaire distributed online through Google Forms. Before survey distribution, ethical clearance had been obtained from the institutional research committee (Approval No. 018/MARS/EC/IV/2026; letter issued on 7 April 2026). Eligible nurses received an invitation containing the survey link and information about the study objectives, voluntary participation, anonymity, confidentiality, and data protection procedures. Before accessing the questionnaire, participants were required to complete an electronic informed consent form and provide an electronic signature indicating their voluntary agreement to participate. Only participants who completed the consent process were able to proceed to the questionnaire. Responses were automatically recorded through the online platform and subsequently screened for completeness before inclusion in the final dataset.

### 2.4. Conceptual Framework

The proposed conceptual framework draws upon Sensemaking Theory as the overarching theoretical perspective for understanding how nurses and healthcare teams interpret clinical information and respond to uncertainty in complex healthcare environments [30]. Within this perspective, patient safety depends not only on structural resources but also on healthcare professionals’ ability to recognize meaningful clinical cues, develop interpretations of evolving situations, and translate previous experiences into appropriate and coordinated actions. Accordingly, this study conceptualizes risk pattern recognition (RPR), safety sensemaking (SSM), and learning from events orientation (LEO) as complementary cognitive safety capabilities that may support adaptive responses among frontline nurses. RPR reflects the recognition of recurring patterns and potential warning signals [31,32], SSM concerns the interpretation and organization of information to develop an understanding of complex safety situations [30,33,34], while LEO reflects the orientation toward learning from patient safety events and applying lessons to clinical practice [35,36,37]. Previous research has highlighted the relevance of sensemaking in understanding how healthcare professionals interpret and respond to patient safety situations [30,33,34] and the importance of learning from patient safety events for translating experience into improvements in clinical practice [35,36,37].

Building on this theoretical perspective, the proposed framework examines the relationships among cognitive safety capabilities, adaptive nursing capabilities, and missed nursing care within the context of remote multicultural hospitals. Specifically, nurse moral resilience (NRM) and interprofessional adaptive coordination (IPC) are positioned as adaptive capabilities through which cognitive safety capabilities may be associated with nursing care delivery. NRM reflects the capability of nurses to preserve or restore professional integrity when facing moral adversity, ethical tensions, and competing obligations in clinical practice [38,39], whereas IPC reflects the capability of healthcare professionals across disciplines to coordinate flexibly and responsively through information exchange, role adjustment, collaborative decision-making, and coordination of tasks in response to changing patient care demands [40,41]. In addition, intercultural communication competence (ITCC) is incorporated as a contextual moderating construct reflecting the multicultural characteristics of healthcare services in South Papua [42,43,44].

**Figure 1:**
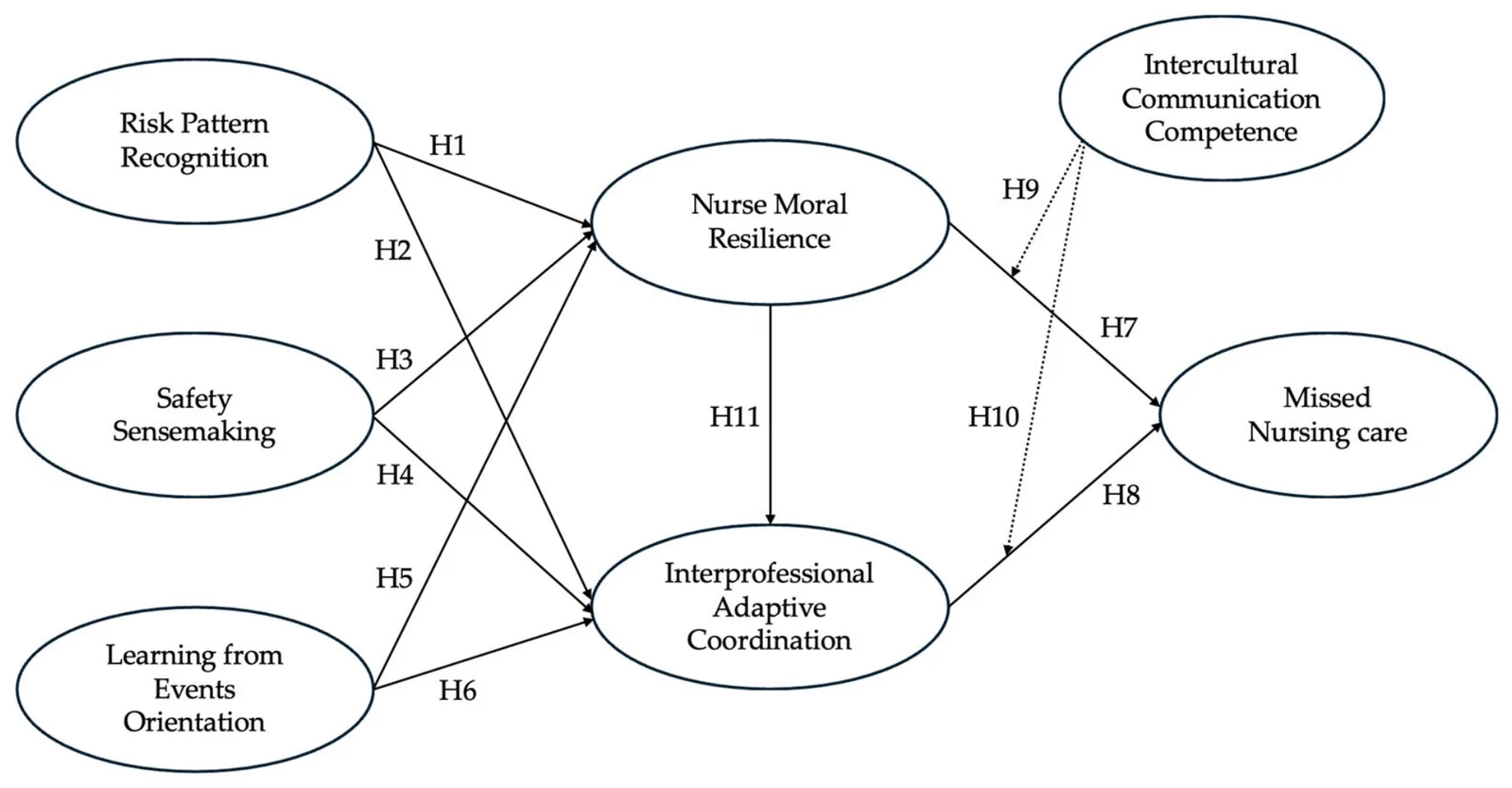
Proposed Conceptual Framework.

Figure 1 presents the proposed conceptual framework that guided the development of the research hypotheses and the subsequent empirical analysis. Figure 1 presents the overall structure of the proposed research model and the hypothesized relationships among the study constructs. To facilitate consistent interpretation of the conceptual model, Table 1 summarizes the conceptual definitions adopted for each construct in the present study. These definitions provide the conceptual foundation for the hypotheses developed in the following section and clarify how each construct is interpreted within the context of patient safety in remote multicultural hospitals.

The measurement items were selected from previously reported instruments and adapted to reflect the context of nursing practice in remote hospitals. The adaptation focused primarily on wording and contextual clarity while retaining the substantive meaning of the original items. The final questionnaire contained the retained indicators. Table 1 provides the conceptual definitions and corresponding sources used to guide the operationalization of each construct.

The conceptualization in Table 1 provides a context-sensitive basis for modelling missed nursing care among frontline nurses in remote and culturally diverse hospitals in South Papua. Rather than viewing missed care through a single individual or organizational lens, the framework integrates cognitive, professional, relational, and cultural dimensions of everyday nursing practice, reflecting the complexity of patient safety work in geographically isolated settings. This conceptualization provides the basis for examining the structural relationships proposed in the following hypotheses.

### 2.5. Hypothesis Development

Building upon the conceptual framework illustrated in Figure 1 and the construct definitions presented in Table 1, the following hypotheses were developed to examine the proposed associations among the study constructs. Each hypothesis was derived from Sensemaking Theory and supported by previous empirical findings in patient safety, nursing resilience, interprofessional collaboration, and healthcare communication.

Within a sensemaking perspective, recognition of safety-relevant cues represents an important cognitive capability for understanding complex clinical situations [31,32]. Nurse moral resilience (NRM), meanwhile, reflects nurses’ capability to preserve or restore professional integrity when facing moral adversity, ethical tensions, and competing obligations. Previous studies have examined moral resilience in relation to moral distress, burnout, second-victim experiences, and resilience within demanding healthcare environments. Gautam et al. (2022) [48], Antonsdottir et al. (2021) [49], Asadi et al. (2025) [50], Rushton et al. (2022) [51], and Wei et al. (2019) [52] provide evidence concerning moral resilience and related adaptive responses among healthcare professionals. Based on this conceptual relationship, the present study proposes a positive structural association between risk pattern recognition and nurse moral resilience.

**H1.**  *Risk Pattern Recognition is positively associated with Nurse Moral Resilience.*

Previous research has emphasized the relevance of interprofessional collaboration and coordinated practice to patient-centered care and patient safety. Gantayet-Mathur et al. (2022) [53] examined patient-centered care and interprofessional collaboration, while Jiang et al. (2024) [54] reviewed interprofessional approaches related to patient safety. Zaheer et al. (2021) [55], Moloro et al. (2025) [56], and Patel et al. (2025) [57] further addressed teamwork, interprofessional collaboration, and competency development in healthcare settings. From a structural perspective, the recognition of safety-relevant information can be considered conceptually related to the capacity to coordinate care across professional roles. Accordingly, the present study proposes a positive structural association between risk pattern recognition and interprofessional adaptive coordination.

**H2.**  *Risk Pattern Recognition is positively associated with Interprofessional Adaptive Coordination.*

Previous research has examined sensemaking as a means of understanding safety-related situations within complex healthcare environments [30,33,34], while more recent research has considered patient safety sensemaking in relation to complexity and the interpretation of healthcare situations [58]. Nurse Moral Resilience reflects the capability to preserve or restore professional integrity when facing moral adversity and ethical tensions. Research on moral resilience has examined its relationship with moral distress, burnout, moral injury, and other challenging professional circumstances among healthcare professionals [48,49,50,51,52]. Taken together, these conceptual perspectives provide a basis for examining whether safety sensemaking is positively associated with nurse moral resilience.

**H3.**  *Safety Sensemaking is positively associated with Nurse Moral Resilience.*

Existing research has emphasized the relevance of interprofessional collaboration, teamwork, and competency development to coordinated healthcare practice and patient safety [53,54,55,56,57]. From a structural perspective, safety sensemaking and interprofessional adaptive coordination represent conceptually related capabilities involving the interpretation and organization of information and the coordination of responses across professional roles. Accordingly, the present study proposes a positive structural association between safety sensemaking and interprofessional adaptive coordination.

**H4.**  *Safety Sensemaking is positively associated with Interprofessional Adaptive Coordination.*

Learning from events orientation (LEO) reflects the extent to which healthcare professionals view patient safety incidents and unexpected clinical events as opportunities for continuous improvement rather than individual blame [35,36,37]. From the perspective of Sensemaking Theory, reflection on previous events is relevant to how healthcare professionals interpret clinical experiences and construct meaning from safety-related situations [30]. Event learning has also been associated with continuous quality improvement and patient safety culture, highlighting the relevance of learning-oriented environments to reflective practice and collective learning [59]. Nurse moral resilience represents the capability to preserve or restore professional integrity when facing moral adversity and ethical tensions [38,39]. Previous research has examined moral resilience in relation to moral distress, burnout, moral injury, and other ethically demanding healthcare circumstances [48,49,50,51,60,61]. These perspectives provide a basis for examining whether a stronger orientation toward learning from events is positively associated with nurse moral resilience.

**H5.**  *Learning From Events Orientation is positively associated with Nurse Moral Resilience.*

Event learning has been linked to continuous quality improvement and patient safety culture [59], while interprofessional research has emphasized the importance of communication, shared understanding, cooperation, and collaborative practice in multidisciplinary healthcare teams [53,54,55,56,57,62]. Bendowska and Baum (2023) [62] further highlighted the significance of cooperation in interdisciplinary healthcare teams. From a structural perspective, an orientation toward learning from clinical events is conceptually related to the development of shared approaches and coordination across professional roles. Accordingly, the present study proposes a positive structural association between Learning From Events Orientation and Interprofessional Adaptive Coordination.

**H6.**  *Learning From Events Orientation is positively associated with Interprofessional Adaptive Coordination.*

Previous studies have examined moral resilience in relation to moral distress, burnout, moral injury, and the challenges experienced by healthcare professionals in demanding clinical environments [48,49,50,51,60,61]. Missed nursing care, in contrast, represents delayed, incomplete, or omitted nursing care activities [3,4,6,45,46]. Recent literature has identified missed or unfinished nursing care as a phenomenon associated with prioritization, care demands, organizational conditions, staffing, and resource adequacy [63,64,65,66,67,68,69]. Research has also linked nursing workforce conditions and patient safety-related capabilities with the delivery and omission of nursing care [70,71]. Within this structural perspective, nurse moral resilience represents an individual adaptive capability that may be associated with differences in nurses’ perceptions of missed nursing care. Therefore, the present study proposes a negative structural association between Nurse Moral Resilience and Missed Nursing Care.

**H7.**  *Nurse Moral Resilience is negatively associated with Missed Nursing Care.*

Interprofessional adaptive coordination reflects the capability of healthcare professionals across disciplines to exchange information, adjust roles, engage in collaborative decision-making, and coordinate care activities [40,41]. Previous research has consistently examined interprofessional collaboration, teamwork, cooperation, and competency development in relation to coordinated healthcare practice and patient safety [53,54,55,56,57,62]. Missed nursing care has meanwhile been associated with care prioritization, unfinished care, patient safety culture, staffing conditions, and resource inadequacy [63,64,65,66,67,68,69]. Studies addressing nursing workforce requirements and care delivery in remote and resource-constrained settings further highlight the relevance of staffing and workforce capacity to nursing care provision [68,69]. Research on nurse burnout and patient safety outcomes [70] and on patient safety competency and adverse-event reporting [71] also underscores the importance of coordinated and safety-oriented clinical practice. From this structural perspective, greater interprofessional adaptive coordination is expected to be negatively associated with perceived missed nursing care.

**H8.**  *Interprofessional Adaptive Coordination is negatively associated with Missed Nursing Care.*

Intercultural Communication Competence (ITCC) refers to nurses’ ability to adapt communication with patients and colleagues across differences in values, beliefs, language, health literacy, expectations, and communication preferences [42,43,44]. Previous research has emphasized the importance of culturally competent healthcare practices and communication skills for understanding and interacting effectively across cultural differences [13,14,15]. Communication-focused studies have further highlighted the relevance of communication education to patient safety [72], identified barriers in communication between healthcare providers and culturally diverse patients [73], and examined structured communication practices in relation to patient safety [74]. Research conducted in Papua has additionally identified cultural barriers as an important consideration in healthcare delivery [75]. In a culturally diverse clinical environment, the association between Nurse Moral Resilience and Missed Nursing Care may therefore differ according to nurses’ level of intercultural communication competence. Accordingly, the present study proposes that Intercultural Communication Competence moderates the association between Nurse Moral Resilience and Missed Nursing Care.

**H9.**  *Intercultural Communication Competence moderates the association between Nurse Moral Resilience and Missed Nursing Care.*

Previous research has emphasized the importance of culturally competent communication in healthcare [15], while studies have also identified communication education, communication barriers, and structured communication practices as relevant considerations for patient safety and collaborative care [72,73,74]. Evidence from Papua further highlights the importance of cultural considerations and communication barriers in healthcare delivery [75]. These perspectives suggest that the strength of the association between interprofessional adaptive coordination and perceived missed nursing care may vary according to nurses’ level of intercultural communication competence. Accordingly, the present study proposes the following hypothesis:

**H10.**  *Intercultural Communication Competence moderates the association between Interprofessional Adaptive Coordination and Missed Nursing Care.*

Research by Gautam et al. (2022) [48], Antonsdottir et al. (2021) [49], Asadi et al. (2025) [50], Rushton et al. (2022) [51], and Wei et al. (2019) [52] has examined moral resilience in relation to demanding clinical and professional environments. Interprofessional collaboration research, meanwhile, emphasizes communication, teamwork, cooperation, and competency development as dimensions of coordinated healthcare practice [53,54,55,56,57,62]. From a structural perspective, moral resilience and interprofessional adaptive coordination represent distinct but potentially complementary professional capabilities: moral resilience concerns nurses’ capacity to maintain professional integrity under adversity, whereas adaptive coordination concerns the capacity to work collaboratively across professional boundaries. On this basis, the present study proposes that Nurse Moral Resilience is positively associated with Interprofessional Adaptive Coordination.

**H11.**  *Nurse Moral Resilience is positively associated with Interprofessional Adaptive CoordinationTop of FormBottom of Form.*

### 2.6. Data Analysis

Further, the survey data were analyzed using Partial Least Squares–Structural Equation Modeling (PLS-SEM) with SmartPLS 4 software (Ringle et al., 2024) [76]. PLS-SEM was employed to estimate the reflective measurement models and the structural model simultaneously and to examine the hypothesized structural associations among the study constructs [29]. The analytical procedure was conducted sequentially, beginning with assessment of the measurement model (outer model), followed by evaluation of the structural model (inner model), hypothesis testing, moderation and mediation analyses where specified, and assessment of out-of-sample predictive performance.

The measurement model (outer model) was first assessed to establish the reliability and validity of the reflective constructs. Indicator reliability was evaluated using standardized outer loadings, while internal consistency reliability was assessed using Cronbach’s alpha, rho_a, and composite reliability. Convergent validity was examined using the average variance extracted (AVE). Discriminant validity was assessed using the heterotrait–monotrait ratio (HTMT), following the criterion proposed by Henseler et al. (2015) [77]. Indicator retention or removal was considered based on statistical performance and conceptual relevance to the respective construct, consistent with established PLS-SEM procedures [29].

Collinearity was subsequently assessed before interpretation of the structural relationships. Full-collinearity variance inflation factor (VIF) values were examined to identify potential collinearity and to assess common method bias using the full-collinearity approach proposed by Kock (2015) [78]. This assessment was used as a diagnostic procedure rather than as evidence of causal relationships among the constructs.

The structural model (inner model) was then evaluated after satisfactory measurement-model assessment. Collinearity among the predictor constructs was examined using VIF values, followed by assessment of standardized path coefficients to determine the direction and magnitude of the hypothesized structural associations. Hypothesis testing was conducted using bootstrapping with 10,000 resamples, employing a one-tailed test at an alpha level of 0.05 in accordance with the directional hypotheses. Statistical inference was based on the resulting path coefficients, *p*-values, and bootstrapped confidence intervals. The explanatory capacity of endogenous constructs was assessed using the coefficient of determination (R^2^), while the relative contribution of predictor constructs was examined using effect size (f^2^) [29].

The direct structural associations specified in H1–H8 and H11 were assessed through the estimated path coefficients and their bootstrapped significance parameters. The moderating associations specified in H9 and H10 were examined through the corresponding interaction terms, with the interpretation based on the sign, magnitude, statistical significance, and confidence interval of the interaction coefficient. These analyses were interpreted as structural associations rather than causal effects, consistent with the relational design of the study.

Where indirect relationships were specified, mediation analysis was conducted by assessing the significance of the indirect effects obtained through bootstrapping. The analysis considered the estimated indirect effect and its bootstrapped statistical inference, with interpretation based on the relationship between the indirect and corresponding direct effects. The mediation analysis followed the PLS-SEM framework proposed by Nitzl et al. (2016) [79], which emphasizes the assessment of indirect effects within the structural model.

Beyond explanatory assessment, the predictive performance of the model was evaluated using the Cross-Validated Predictive Ability Test (CVPAT). CVPAT was used to compare the model’s cross-validated prediction errors with those of an appropriate benchmark model [80]. The analysis followed the methodological extensions and reporting recommendations proposed by Sharma et al. (2023) [81]. The resulting average loss difference and associated statistical assessment were considered to determine whether the proposed model demonstrated predictive performance relative to the benchmark. CVPAT was therefore used as a complementary assessment of predictive ability rather than as evidence of causal relationships.

Overall, the analytical procedure integrated outer-model assessment, inner-model evaluation, directional hypothesis testing, moderation analysis, mediation analysis where specified, and out-of-sample predictive assessment using CVPAT. The procedures were designed to evaluate the reliability and validity of the measurement instruments, the strength and direction of the proposed structural associations, the explanatory capacity of the model, and its predictive performance within the study context.

## 3. Results

### 3.1. Participants’ Characteristics

Of the 338 nurses who met the eligibility criteria, 320 returned completed questionnaires, corresponding to a response rate of 94.7%. After eight questionnaires were excluded because of incomplete responses, 312 questionnaires were retained for the final analysis. As shown in Table 2, the resulting sample represented a relatively experienced frontline nursing workforce, with participants distributed across different age groups, educational backgrounds, lengths of clinical experience, employment arrangements, ethnic backgrounds, and clinical work units. This diversity provides an appropriate empirical context for examining the proposed relationships among individual safety capabilities, interprofessional coordination, intercultural communication competence, and perceived missed nursing care.

The participants were predominantly composed of nurses in the early-to-mid stages of their professional careers, although a substantial proportion had more than six years of clinical experience. This distribution indicates that the sample was not concentrated exclusively among newly qualified nurses and included participants with accumulated clinical experience. Such variation is relevant to the study context because several constructs in the proposed framework, particularly risk pattern recognition, safety sensemaking, learning from events, and moral resilience, concern nurses’ perceptions and responses developed through clinical practice.

The ethnic composition is particularly relevant to the study setting. Indigenous Papuan nurses constituted the majority of respondents, while non-Indigenous Papuan nurses also represented a substantial proportion of the sample. Thus, the participating workforce included nurses from different cultural backgrounds within the same hospital environment. This characteristic is pertinent to the interpretation of intercultural communication competence as an individual-level capability in patient-facing communication. However, the descriptive composition of the sample should not be interpreted as evidence of differences between ethnic groups, as no subgroup comparison was specified in the present structural model.

Participants were drawn from all four participating hospitals, with the largest proportions contributed by RSUD Merauke and RS Bunda Pengharapan, while the two military hospitals contributed smaller but substantive groups of respondents. The sample also covered multiple clinical environments, including inpatient wards, emergency care, intensive care, operating rooms, and haemodialysis services. This distribution provides variation in the clinical settings represented in the dataset, rather than concentrating the observations within a single type of nursing service. The distribution across hospitals was 117 nurses from RSUD Merauke, 98 from RS Bunda Pengharapan, 51 from RS Tentara Angkatan Darat Merauke, and 46 from RS Angkatan Laut Merauke.

### 3.2. Outer Model (Measurement Model)

The measurement model was assessed to evaluate indicator reliability, internal consistency reliability, convergent validity, and discriminant validity following the recommended procedures for PLS-SEM [29]. Indicator reliability was examined using outer loadings, while internal consistency reliability and convergent validity were assessed using Cronbach’s alpha (CA), rho_a, composite reliability (CR), and average variance extracted (AVE). Further, discriminant validity was assessed using the heterotrait–monotrait ratio (HTMT).

#### 3.2.1. Indicator Reliability and Convergent Validity

As presented in Table 3, the initial assessment of the six candidate indicators resulted in the removal of one indicator from the missed nursing care (MNC) construct. MNC5, “I have occasionally skipped the hand-washing procedure before and after caring for patients,” showed an outer loading below 0.60 and therefore did not meet the indicator-retention criterion adopted in this study [29]. Although the item represents a specific form of missed nursing care involving the omission of an infection-prevention practice, its content is narrower than the broader forms and circumstances of delayed, incomplete, or omitted nursing care represented by the retained indicators. The combination of its relatively weak empirical representation and narrower conceptual focus provided the basis for its removal. This decision concerned the measurement performance and content breadth of the indicator and did not imply that hand hygiene is clinically unimportant. No other indicator was removed during the final measurement assessment.

Following the removal of MNC5, the outer loadings of the retained indicators ranged from 0.631 to 0.925. Although several indicators had loadings below the preferred value of 0.708, all retained indicators exceeded 0.60 and were therefore considered acceptable for retention in the context of the overall measurement model [29]. Their retention was further supported by the satisfactory internal consistency reliability and convergent validity of the corresponding constructs. For the MNC construct, the final five-indicator measurement model demonstrated strong internal consistency, with Cronbach’s α = 0.929, rho_a = 0.936, and composite reliability = 0.947, while the AVE of 0.780 indicated that the construct accounted for a substantial proportion of the variance in its retained indicators. The final measurement model was therefore retained without further indicator deletion, providing an adequate basis for subsequent structural model assessment.

**Table 3:** Outer Loading, Reliability and Validity.

Variable	Code	Indicator	Mean	OL
Risk Pattern Recognition	RPR1	I am able to recognize risks that could jeopardize patient safety.	4.069	0.723
	RPR2	Patient safety information from other units helps me anticipate the risk of errors occurring in my unit.	4.181	0.686
	RPR3	Standardized work procedures help me recognize potential errors in nursing care.	4.069	0.683
	RPR4	Experience from previous patient mishaps helps me recognize similar risks in the future.	4.155	0.741
	RPR5	The incident reporting system helps me recognize recurring patient safety risks.	4.249	0.801
	RPR6	My supervisor reminded me that it is important to assess patient safety risks from the moment I enter the hospital.	4.123	0.784
CA = 0.836, rho_a = 0.849, rho_c = 0.877, AVE = 0.544				
Safety Sensemaking	SSM1	I was helped by guidance from my superiors in explaining the rationale for each patient safety decision.	4.310	0.761
	SSM2	I was encouraged to make decisions quickly when faced with emergency situations.	4.090	0.840
	SSM3	Team discussions helped me understand patient safety risks more clearly.	3.917	0.694
	SSM4	Explanations of SOPs in my unit helped me clearly assess patient conditions.	3.845	0.780
CA = 0.770, rho_a = 0.777, rho_c = 0.853, AVE = 0.594				
Learning From Events Orientation	LEO1	I learn from previous incidents to improve nursing care without blaming colleagues.	4.022	0.631
	LEO2	I can learn from mistakes made in care to improve nursing practice.	3.787	0.754
	LEO3	I will discuss patient complaints about nursing care in team discussions.	3.354	0.742
	LEO4	I actively participate in patient safety training to prevent similar incidents in the future.	3.740	0.704
	LEO5	I can remember past mistakes as learning materials in our unit.	3.487	0.702
	LEO6	I can see the documentation related to the error that occurred	3.632	0.794
CA = 0.817, rho_a = 0.818, rho_c = 0.867, AVE = 0.523				
Nurse Moral Resilience	NRM1	I adhere to my professional values when facing pressure at work.	3.968	0.705
	NRM2	I maintain a clear mind to know what is right and wrong when dealing with stress at work.	3.859	0.661
	NRM3	I am able to articulate the rationale for actions taken in emergency situations.	4.029	0.855
	NRM4	Even if I am threatened by a patient’s family, it does not diminish my commitment to patient safety.	3.794	0.784
	NRM5	I can maintain the correct attitude even after being instructed by my superiors.	3.852	0.811
	NRM6	Understanding what is right helps me manage a heavy workload.	4.101	0.801
CA = 0.863, rho_a = 0.874, rho_c = 0.898, AVE = 0.597				
Interprofessional Adaptive Coordination	IPC1	My team involves patients in helping determine treatment goals.	4.097	0.664
	IPC2	Coordination between the physician and nurses is effective.	4.166	0.863
	IPC3	Coordination is routinely conducted in the hospital team members communicate well with each other	4.148	0.845
	IPC4	The physician in charge of the patient and the nurse do not blame each other when a patient complains about poor service.	4.141	0.855
CA = 0.822, rho_a = 0.824, rho_c = 0.884, AVE = 0.658				
Intercultural Communication Competence	ITCC1	I am able to communicate with patients from different cultural or ethnic backgrounds.	4.123	0.893
	ITCC2	I can respect differences in patient beliefs, ethnics or religions.	4.076	0.848
	ITCC3	I am able to avoid using words that offend patients based on their backgrounds.	4.087	0.827
	ITCC4	I feel I can relate better to patients if I understand their customs.	4.191	0.704
CA = 0.841, rho_a = 0.879, rho_c = 0.891, AVE = 0.674				
Missed Nursing Care	MNC1	Assessment of the patient’s basic needs was sometimes delayed.	4.556	0.886
	MNC2	Patient safety assessments were incomplete.	4.635	0.925
	MNC3	I neglected the patient’s emotional state.	4.596	0.879
	MNC4	Patient privacy is sometimes not consistently maintained.	4.606	0.896
	MNC6	Limited resources led to missed nursing interventions.	4.527	0.827
CA = 0.929, rho_a = 0.936, rho_c = 0.947, AVE = 0.780				

CA = Cronbach’s alpha, OL = outer loading, AVE = average variance extracted.

In addition to these measurement properties, the relatively high mean scores of the retained MNC indicators warranted examination of their response distributions. As reported in Supplementary Table S1, responses were concentrated toward the upper end of the scale, with 62.1–70.0% of respondents selecting the highest response category across the five retained indicators. Nevertheless, observable variation remained, with standard deviations ranging from 0.589 to 0.693 and observed ranges of 3–5 for MNC1–MNC4 and 2–5 for MNC6. These distributional characteristics indicate upper-end response concentration and potentially restricted variability and should therefore be considered when interpreting the structural associations involving MNC.

As shown in Table 3, CA values ranged from 0.770 to 0.929, rho_a values ranged from 0.777 to 0.936, and CR values ranged from 0.853 to 0.947. All values exceeded the recommended threshold of 0.70 [29]. The AVE values ranged from 0.523 to 0.780, with all constructs exceeding the recommended minimum of 0.50, thereby supporting convergent validity [29]. These results indicate that the retained indicators demonstrated acceptable reliability and convergent validity for the subsequent structural model assessment.

#### 3.2.2. Discriminant Validity

Discriminant validity was assessed using the heterotrait–monotrait ratio (HTMT). As presented in Table 4, the HTMT values among the substantive constructs ranged from 0.046 to 0.892. All observed HTMT values were below the recommended threshold of 0.90, indicating acceptable discriminant validity based on the prespecified criterion [77]. The highest observed HTMT value was between intercultural communication competence (ITCC) and interprofessional adaptive coordination (IPC) (HTMT = 0.892). The bootstrap bounds for this estimate ranged from 0.844 to 0.953 (5th–95th percentiles). Although the observed HTMT value was below 0.90, the upper bootstrap bound exceeded this threshold, indicating uncertainty around the estimate and warranting caution in interpreting discriminant validity between ITCC and IPC. The bootstrap interval did not include 1.000; however, this is a separate observation and does not indicate that the entire interval falls below the 0.90 criterion. Accordingly, ITCC and IPC were distinguishable based on the observed HTMT value, while the bootstrap results indicate that their discriminant validity should be interpreted cautiously.

The interaction terms, ITCC × NRM and ITCC × IPC, were also included in the measurement assessment as model interaction constructs. Their HTMT values did not indicate problematic overlap with the substantive constructs. The highest value involving the interaction terms was observed between ITCC × NRM and MNC (0.573) and between ITCC × IPC and MNC (0.577), remaining below the adopted threshold. The retained measurement model demonstrated acceptable indicator reliability, internal consistency reliability, and convergent validity across the study constructs.

The measurement model demonstrated acceptable reliability, convergent validity, and discriminant validity. All retained indicators met the adopted outer-loading criteria, while Cronbach’s alpha, rho_a, and composite reliability exceeded 0.70, and AVE values exceeded 0.50. On this basis, the measurement model was considered adequate for proceeding to the structural model assessment.

### 3.3. Inner Model (Structural Model)

#### 3.3.1. Collinearity Assessment

Collinearity among the predictor constructs was assessed using the inner variance inflation factor (VIF). All inner VIF values were below the recommended thresholds, indicating that critical collinearity was not present among the predictor constructs in the structural model [29]. The VIF results also provide a diagnostic assessment relevant to potential common method bias (CMB). Specifically, all inner VIF values were below the conservative threshold of 3.0 and the more stringent criterion of 3.3 proposed for full-collinearity assessment [78]. These findings do not indicate problematic collinearity or severe common method bias in the estimated structural relationships. However, the VIF results should be interpreted as diagnostic evidence rather than as definitive proof that common method bias is absent. The structural model was therefore considered suitable for subsequent assessment of explanatory power, predictive performance, and hypothesized relationships.

#### 3.3.2. Explanatory Power

**Figure 2:**
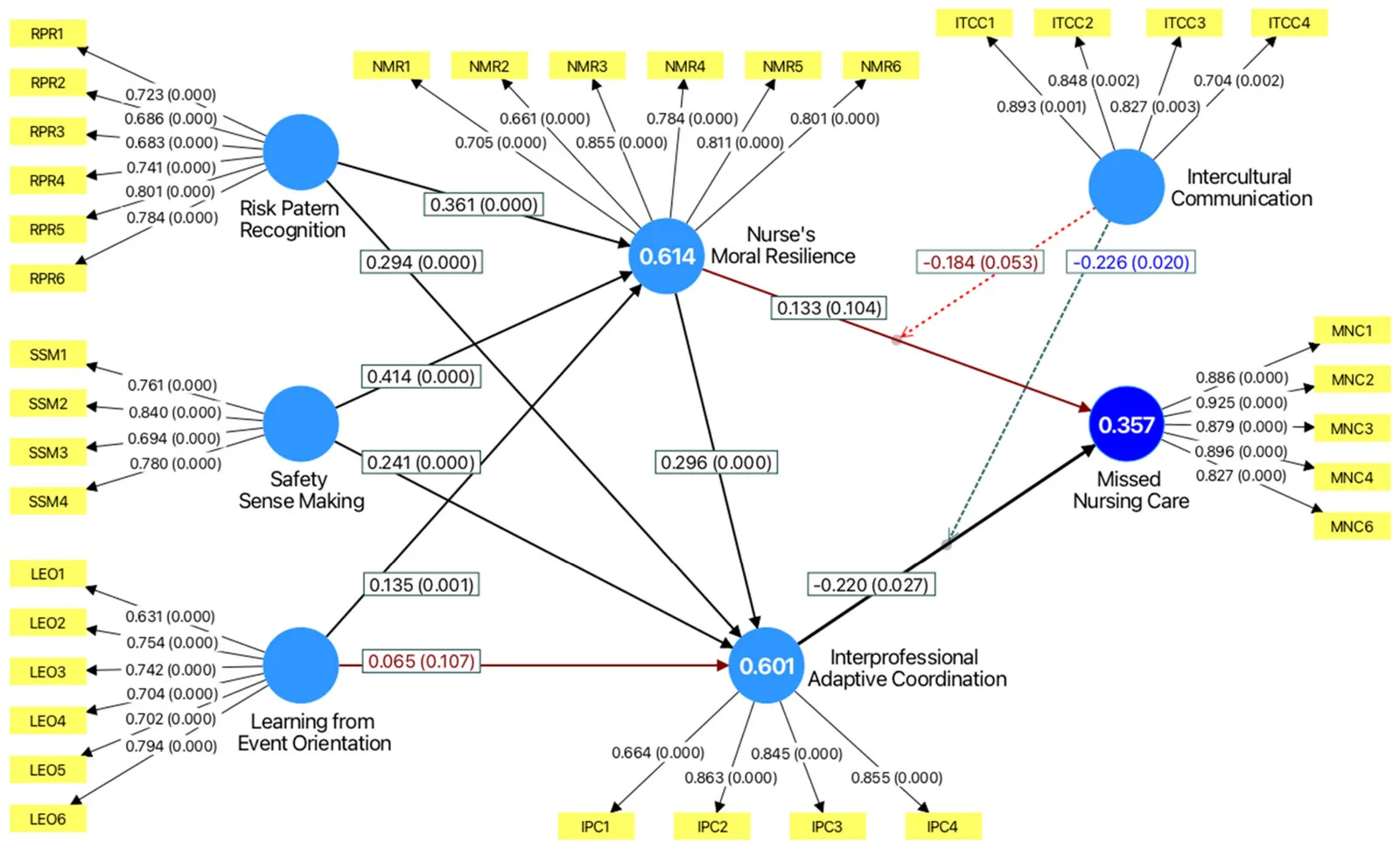
Inner Model (Structural Model).

The structural model evaluation showed that the model explained 61.4% of the variance in nurse moral resilience (R^2^ = 0.614) and 60.1% of the variance in interprofessional adaptive coordination (R^2^ = 0.601). Missed nursing care had an R^2^ value of 0.357, indicating that the constructs included in the structural model jointly accounted for 35.7% of the variance in perceived missed nursing care. The remaining variance was not explained by the constructs included in the present model and may reflect other factors that were not examined in this study (Figure 2).

The effect size (f^2^) results indicated that the largest individual effects were observed for the relationships between safety sensemaking and nurse moral resilience (f^2^ = 0.233) and between risk pattern recognition and nurse moral resilience (f^2^ = 0.194), both representing moderate effect sizes. The remaining relationships showed smaller effect sizes, including the interaction effects of intercultural communication competence on the associations of interprofessional adaptive coordination with missed nursing care (f^2^ = 0.059) and nurse moral resilience with missed nursing care (f^2^ = 0.031). These results indicate that the relative contribution of individual predictor relationships varied across the endogenous constructs, with the largest effects observed for the cognitive safety capability relationships involving nurse moral resilience.

#### 3.3.3. Predictive Power

Predictive performance was assessed using PLS-Predict and the Cross-Validated Predictive Ability Test (CVPAT) to evaluate the model’s out-of-sample predictive performance [80,81]. The PLS-Predict results indicated that all endogenous constructs had Q^2^_predict values greater than zero, providing evidence of predictive relevance. CVPAT was subsequently used to statistically compare the predictive performance of the PLS-SEM model with two benchmark approaches: the indicator average (IA) and the linear model (LM). In CVPAT, a negative average loss difference indicates that the PLS-SEM model produces a lower prediction error than the corresponding benchmark [81].

As shown in Table 5, the overall model produced a significantly lower prediction loss than both benchmark approaches. Compared with the indicator-average benchmark, the PLS-SEM model yielded an average loss difference of −0.107 (*p* < 0.001), while the comparison with the linear model produced an average loss difference of −0.039 (*p* < 0.001). These results indicate that, at the overall model level, the proposed PLS-SEM model achieved significantly better out-of-sample predictive performance than both the naïve indicator-average benchmark and the linear-model benchmark.

The construct-level results showed some variation in predictive performance across the endogenous constructs. Compared with the IA benchmark, PLS-SEM produced significantly lower prediction loss for interprofessional adaptive coordination (−0.130, *p* < 0.001), nurse moral resilience (−0.102, *p* < 0.001), and missed nursing care (−0.083, *p* < 0.001). Compared with the LM benchmark, PLS-SEM also produced significantly lower prediction loss for interprofessional adaptive coordination (−0.026, *p* < 0.001) and missed nursing care (−0.110, *p* < 0.001). For nurse moral resilience, however, the average loss difference was positive (0.031, *p* = 0.044), indicating that the LM benchmark produced lower prediction loss for this construct. Comprehensively, these construct-level findings show that the predictive advantage of PLS-SEM was not uniform across all endogenous constructs when compared with the LM benchmark. At the overall model level, however, PLS-SEM demonstrated significantly lower prediction loss than both the IA and LM benchmarks, as indicated by the negative average loss differences of −0.107 (*p* < 0.001) and −0.039 (*p* < 0.001), respectively.

#### 3.3.4. Hypothesis Testing

Table 6 presents the results of hypothesis testing based on the PLS-SEM structural model. The significance of the hypothesized relationships was assessed using bootstrapping with 10,000 subsamples and a significance level of 0.05. Hypothesis decisions were based on the estimated path coefficients, *p*-values, and corresponding confidence intervals.

For nurse moral resilience, risk pattern recognition, safety sensemaking, and learning from events orientation showed significant positive associations. Risk pattern recognition was positively associated with nurse moral resilience (β = 0.361, *p* < 0.001), supporting H1. Safety sensemaking also showed a significant positive association with nurse moral resilience (β = 0.414, *p* < 0.001), supporting H3. Learning from events orientation was positively associated with nurse moral resilience (β = 0.135, *p* = 0.001), supporting H5. Among these antecedents, safety sensemaking had the largest standardized path coefficient, followed by risk pattern recognition and learning from events orientation.

For interprofessional adaptive coordination, risk pattern recognition (β = 0.294, *p* < 0.001), safety sensemaking (β = 0.241, *p* < 0.001), and nurse moral resilience (β = 0.296, *p* < 0.001) showed significant positive associations, supporting H2, H4, and H11, respectively. In contrast, learning from events orientation was not significantly associated with interprofessional adaptive coordination (β = 0.065, *p* = 0.107); therefore, H6 was not supported.

For missed nursing care, interprofessional adaptive coordination showed a significant negative association with missed nursing care (β = −0.220, *p* = 0.027), supporting H8. Nurse moral resilience was not significantly associated with missed nursing care (β = 0.133, *p* = 0.104); therefore, H7 was not supported. The observed coefficient was positive, rather than negative as hypothesized.

The moderation analysis produced different results for the two interaction terms. The interaction between intercultural communication competence and nurse moral resilience was not statistically significant (β = −0.184, *p* = 0.053); therefore, H9 was not supported. In contrast, the interaction between intercultural communication competence and interprofessional adaptive coordination was statistically significant (β = −0.226, *p* = 0.020), supporting H10.

The primary hypothesis-testing framework applied one-tailed significance testing to the prespecified directional hypotheses. Given that H9 and H10 were formulated as non-directional moderation hypotheses, their statistical conclusions were additionally assessed using two-tailed significance levels. The ITCC × NMR interaction remained non-significant (β = −0.184, two-tailed *p* = 0.106), whereas the ITCC × IPC interaction remained statistically significant (β = −0.226, two-tailed *p* = 0.041). Thus, the inferential conclusions for both moderation hypotheses remained unchanged: H9 was not supported, whereas H10 remained supported.

Because MNC6 differs from the other retained MNC indicators by explicitly attributing missed nursing interventions to limited resources, a sensitivity analysis was conducted to assess whether its inclusion materially influenced the principal structural findings involving MNC. The model was re-estimated after excluding MNC6 while retaining the original structural specification. As reported in Supplementary Table S2, the IPC–MNC association remained negative and statistically significant, although its magnitude was slightly attenuated from β = −0.220 (*p* = 0.027) in the original model to β = −0.193 (*p* = 0.041) after excluding MNC6. The ITCC × IPC interaction likewise remained negative and statistically significant, changing only slightly from β = −0.226 (*p* = 0.020) to β = −0.239 (*p* = 0.019). Overall, the direction and statistical conclusions of both associations were maintained after the exclusion of MNC6, with only modest changes in their estimated magnitudes.

#### 3.3.5. Mediation and Moderation Analysis

The specific indirect associations were examined to assess whether the proposed antecedent constructs were indirectly associated with missed nursing care (MNC) through nurse moral resilience (NRM) and interprofessional adaptive coordination (IPC). As shown in Table 7, three specific indirect associations were statistically significant. Risk pattern recognition (RPR) was negatively and indirectly associated with MNC through IPC (β = −0.065, *p* = 0.043), and safety sensemaking (SSM) was likewise negatively and indirectly associated with MNC through IPC (β = −0.053, *p* = 0.040). Nurse moral resilience also showed a significant negative indirect association with MNC through IPC (β = −0.065, *p* = 0.046). The remaining specific indirect associations were not statistically significant (*p* > 0.05).

The pattern of indirect associations indicated that the statistically significant indirect paths were those involving IPC as the intervening construct. Specifically, significant indirect associations were observed for RPR → IPC → MNC, SSM → IPC → MNC, and NRM → IPC → MNC, whereas the indirect paths involving NRM between RPR and MNC, NRM between SSM and MNC, and LEO through either NRM or IPC were not statistically significant. These findings are therefore interpreted as evidence of statistically significant indirect structural associations within the estimated model, rather than as evidence of causal mediation [79].

**Figure 3:**
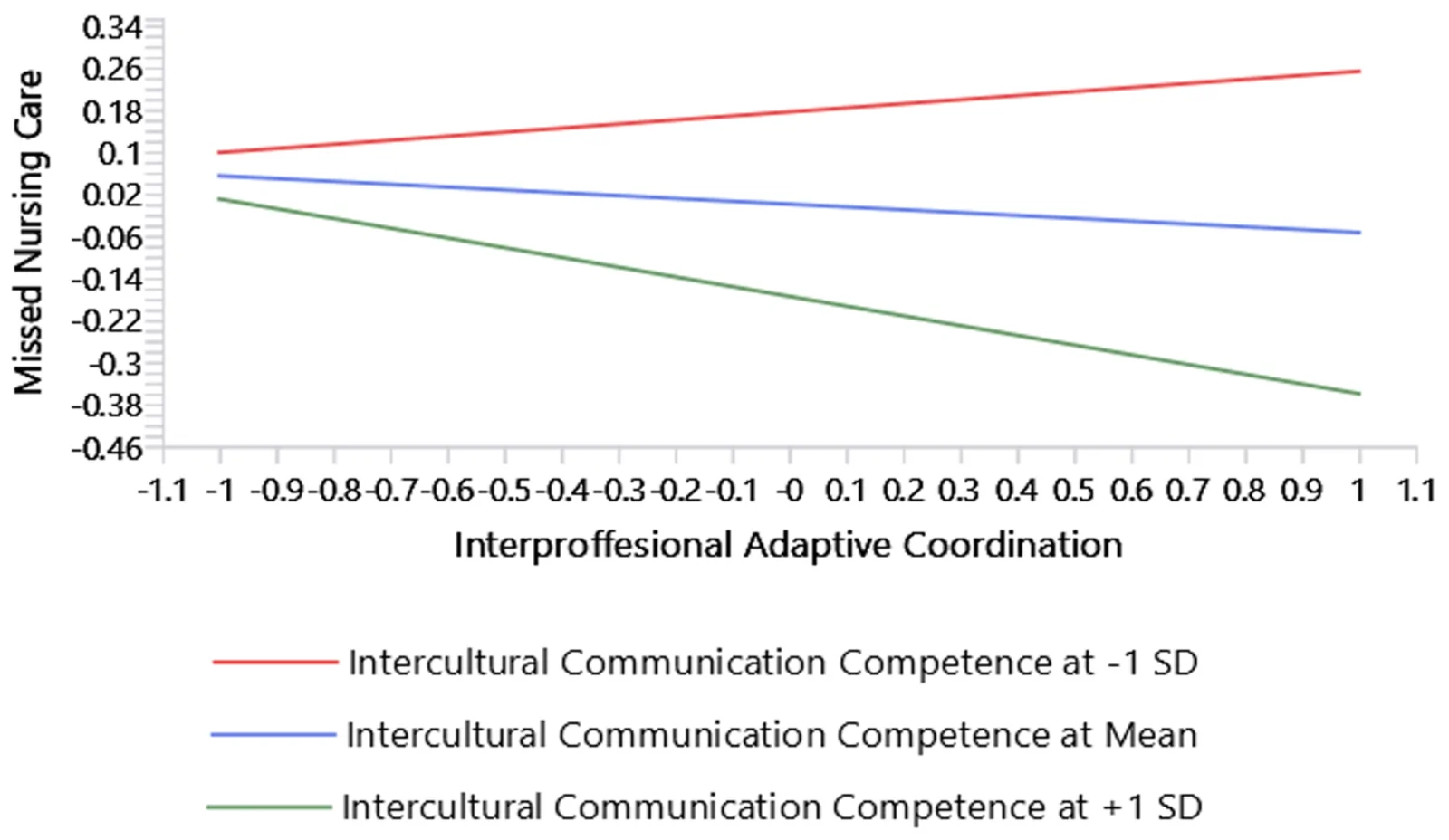
Simple Slope of Moderating Effect of Intercultural Communication Competence.

In addition to the mediation analysis, the significant interaction effect identified in the structural model was further examined using simple slope analysis. This analysis was conducted to visualize the pattern of the interaction between Intercultural communication competence and interprofessional adaptive coordination across different levels of the moderator. The results are presented in Figure 3.

The significant interaction between Intercultural Communication Competence (ITCC) and Interprofessional Adaptive Coordination (IPC) in relation to Missed Nursing Care (MNC) was further examined using a simple-slope analysis (Figure 3). The interaction term was statistically significant (β = −0.226, *p* = 0.020). The figure displays the IPC–MNC association at three levels of ITCC: −1 SD, the mean, and +1 SD. At −1 SD ITCC, the red line shows a relatively flat positive slope, indicating a weak positive association between IPC and MNC at a lower level of ITCC. At the mean level of ITCC, the blue line shows a slightly negative slope, whereas at +1 SD ITCC, the green line shows a substantially steeper negative slope, indicating a stronger negative IPC–MNC association at a higher level of ITCC. Overall, the interaction pattern indicates that the IPC–MNC association became increasingly negative as ITCC increased, while the slope remained relatively flat at the lower level of ITCC.

The conditional-effect estimates provide statistical support for the pattern shown in Figure 3. As reported in Table 8, at low ITCC (−1 SD), the conditional association between IPC and MNC was positive but not statistically significant (β = 0.006, *p* = 0.484), indicating that the positive slope observed in Figure 3 was not statistically distinguishable from zero. At the mean level of ITCC, the conditional IPC–MNC association was negative and statistically significant (β = −0.220, *p* = 0.027). At high ITCC (+1 SD), the association was more strongly negative and statistically significant (β = −0.447, *p* = 0.004). These estimates show that the direction and magnitude of the IPC–MNC association varied across levels of ITCC, with no statistically significant association at the lower ITCC level and increasingly negative associations at the mean and higher ITCC levels. This pattern indicates that higher levels of ITCC were associated with a stronger negative IPC–MNC relationship within the observed sample. The conditional-effect results therefore clarify the statistical pattern illustrated in Figure 3 without implying that ITCC or IPC causally changes the level of missed nursing care.

## 4. Discussion

### 4.1. Cognitive Safety Capabilities in Relation to Nurse Moral Resilience

The three cognitive safety capabilities examined in this study, risk pattern recognition (RPR), safety sensemaking (SSM), and learning from events orientation (LEO), were positively associated with nurse moral resilience (NRM). The convergence of these three significant associations provides empirical support for viewing nurse moral resilience in relation to multiple forms of engagement with patient safety, rather than solely as an individual psychological capacity. This pattern is broadly consistent with previous research linking sensemaking, safety learning, and professional adaptation in complex healthcare environments [30,31,32,33,34,35,36,37,48,49,50,51,52]. More specifically, the results suggest that the cognitive dimensions represented by recognizing safety-relevant patterns, making sense of uncertain situations, and reflecting on safety events are each statistically related to NRM, although the relative strength of these associations differs across the three capabilities. The comparatively stronger association observed for SSM is particularly noteworthy, as it points to interpretation and meaning-making as the dimension most closely related to NRM within the estimated model. This finding adds nuance to earlier work on moral resilience, which has predominantly emphasized moral distress, burnout, moral injury, and other forms of professional adversity [48,49,50,51,52]. Rather than positioning cognitive safety capabilities as determinants of moral resilience, the present findings indicate that these capabilities and NRM are meaningfully related within the same structural context.

Positive association between RPR and NRM suggests that nurses reporting greater engagement in recognizing recurring safety patterns and warning signals also tended to report greater moral resilience. This pattern is compatible with research examining moral resilience in demanding clinical environments characterized by moral distress, burnout, moral injury, and other forms of professional adversity [48,49,50,51,52]. In this context, RPR may be understood primarily as a form of attentional and interpretive engagement with the clinical environment, while NRM reflects an individual capacity concerning professional integrity under difficult circumstances. The two constructs are therefore related at the level of nurses’ engagement with challenging practice situations, although the present cross-sectional data do not establish whether one precedes or influences the other.

Safety sensemaking (SSM) displayed the strongest positive association with NRM among the three cognitive safety capabilities. This pattern is noteworthy because SSM involves making sense of complex, ambiguous, or uncertain safety situations rather than merely noticing individual warning signals [30,33,34]. The association is also compatible with literature describing moral resilience in relation to difficult professional circumstances in which nurses must contend with competing demands and ethical tensions [48,49,50,51,52]. The stronger coefficient for SSM should not be interpreted as evidence that sensemaking has a greater causal role in moral resilience. Rather, within the estimated model, the result indicates a closer statistical association between these two constructs than between NRM and the other cognitive safety capabilities.

The positive association between LEO and NRM provides a related but distinct pattern. LEO concerns the extent to which safety events and unexpected clinical experiences are regarded in terms of reflection, shared learning, and continuous improvement [35,36,37]. Previous research has connected event learning with patient safety culture and continuous quality improvement [59], whereas research on NRM has largely examined moral distress, burnout, moral injury, and resilience in challenging healthcare environments [48,49,50,51,52]. The present finding places these two areas in the same structural model and suggests that nurses who report a stronger orientation toward learning from events also tend to report greater moral resilience. This interpretation remains associational; it does not establish that learning from events produces, strengthens, or sustains moral resilience.

Across the three associations, the most distinctive pattern is the relative strength of SSM compared with RPR and LEO. This may be relevant to the conceptual positioning of cognitive safety capabilities because recognition, interpretation, and learning represent related but non-identical forms of engagement with patient safety experiences [30,31,32,33,34,35,36,37]. At the same time, the findings should be read within the empirical boundaries of this study. The use of cross-sectional, self-reported data and purposive sampling does not permit temporal ordering, causal inference, or generalization beyond the participating setting. The observed associations therefore provide contextual evidence of statistical relationships among cognitive safety capabilities and nurse moral resilience within the study population, without establishing temporal ordering between the constructs.

The relatively high MNC scores observed in this study warrant consideration within the broader evidence on missed nursing care. Direct numerical comparison should be approached cautiously because MNC instruments, response formats, definitions, and reporting practices vary substantially across studies. In a systematic review of acute-care hospitals in low- and middle-income countries, Imam et al. reported MNC prevalence ranging from 15.2% to 86.0% and identified inadequate personnel as the most prominent reported reason for missed care [82]. Similarly, Gong et al., in a systematic review and meta-analysis involving 20,768 nurses from 121 hospitals across 14 countries, reported a wide prevalence range of 6.8–98.1% (median 56.4%), with increased patient volume or acuity, inadequate staffing, and urgent patient situations among the most frequently reported reasons [83]. These findings do not provide a direct benchmark for the scores observed in the present study, but they demonstrate that substantial levels of missed nursing care have been documented across diverse healthcare settings and that resource- and workload-related constraints are frequently reported alongside this phenomenon. Accordingly, the pronounced upper-end responses observed here may be understood within this broader context, while remaining specific to the agreement-based measurement and hospital setting used in the present study.

### 4.2. Adaptive Coordination in Relation to Missed Nursing Care

The pattern of associations involving interprofessional adaptive coordination (IPC) was not uniform across the cognitive and adaptive capabilities examined in the model. Risk pattern recognition (RPR), safety sensemaking (SSM), and nurse moral resilience (NRM) were positively associated with IPC, whereas learning from events orientation (LEO) was not statistically associated with it. This pattern distinguishes immediate engagement with safety-related situations from learning-oriented engagement with previous events. The associations of RPR and SSM with IPC are consistent with literature emphasizing communication, shared understanding, teamwork, and interprofessional collaboration in patient safety [53,54,55,56,57,62]. In contrast, the absence of a statistically significant LEO–IPC association suggests that an orientation toward learning from events is not necessarily accompanied by a corresponding association with nurses’ reported experience of interprofessional coordination.

The stronger associations of RPR and SSM with IPC are particularly relevant to the nature of coordination in complex clinical work. Recognition of safety-relevant cues and interpretation of uncertain situations are closely connected to how nurses perceive information, priorities, and changing care demands during practice [30,31,32,33,34]. Previous studies likewise emphasize communication, shared understanding, and collaborative decision-making as important features of interprofessional practice [53,54,55,56,57]. Within the South Papua setting, these relationships may be especially relevant because nurses work across geographically remote and culturally diverse environments, where differences in local context and communication practices are part of everyday care [9,10,11,12]. The present data, however, do not establish that recognition or sensemaking precedes or produces better coordination; they indicate that nurses reporting higher levels of these capabilities also tended to report higher levels of IPC.

The non-significant association between LEO and IPC offers an important qualification to this pattern. Learning from safety events is closely related to reflection, event analysis, and organizational learning [35,36,37,59], whereas IPC concerns the day-to-day coordination of work across professional boundaries [40,41]. These processes may therefore be related without being interchangeable. In particular, learning from an event may remain at the level of individual reflection or organizational learning unless there are opportunities for shared discussion, leadership engagement, and translation of lessons into team routines. Previous research on interprofessional collaboration also emphasizes the importance of communication structures, cooperation, and opportunities for collective practice [53,54,55,56,57,62]. In remote hospitals in South Papua, where workforce availability and organizational capacity may vary across institutions [9,68,69], the absence of a significant LEO–IPC association may therefore be consistent with a distinction between learning from events and the everyday coordination of clinical work.

The significant negative association between IPC and perceived Missed Nursing Care (MNC) provides a different part of the model’s pattern. Previous studies have repeatedly linked missed or unfinished nursing care with teamwork, communication, patient safety culture, staffing, workload, and resource adequacy [63,64,65,66,67,68,69]. The present finding is consistent with that literature in showing that nurses’ perceptions of interprofessional coordination and missed nursing care are statistically related. At the same time, IPC in this study was assessed through individual nurses’ reports and should therefore be interpreted as a perceived coordination capability at the individual reporting level, rather than as an objectively measured team-level property. This distinction is important because respondents were nested within four hospitals and multiple clinical settings, while institutional clustering was not explicitly modeled. The observed IPC–MNC association should consequently be understood within the perceptions reported by the participating nurses rather than generalized as a characteristic of teams or hospitals as a whole.

The indirect associations provide a further point of interpretation. Specific indirect associations were statistically significant for RPR through IPC, SSM through IPC, and NRM through IPC, while the corresponding indirect paths involving NRM between RPR or SSM and MNC, and those involving LEO through either intermediary, were not statistically significant. Consistent with the PLS-SEM mediation framework, these results indicate statistically supported indirect structural associations within the estimated model [79]. The pattern is notable because IPC appears in each of the significant indirect pathways involving MNC. This does not establish IPC as a causal mechanism; rather, it indicates that part of the statistical association between RPR, SSM, or NRM and perceived MNC is represented through IPC in the specified model.

The NRM–IPC–MNC pathway warrants particular caution because the direct NRM–MNC association was positive but not statistically significant (β = 0.133, *p* = 0.104), whereas the indirect association through IPC was negative and statistically significant. This combination should not be interpreted as evidence of suppression or another specific statistical mechanism without additional analysis. Differences in self-reported perceptions, common-source reporting, measurement characteristics, and unmeasured organizational conditions could all be relevant, but the present cross-sectional data cannot distinguish among these possibilities. The more defensible reading is that NRM was positively associated with IPC, IPC was negatively associated with perceived MNC, and the resulting indirect association between NRM and MNC through IPC was statistically significant within the estimated model [38,39,40,41,81]. This distinction is particularly important because the direct and indirect associations do not show the same direction.

The South Papua context adds relevance to these findings without providing a basis for attributing the observed associations to geography or resource conditions alone. Research from remote areas of Indonesia has documented challenges related to nurse retention and the realities of working in geographically isolated settings [9], while studies of Papua have highlighted the importance of local cultural context in healthcare interactions [10,11,75]. In parallel, international literature identifies staffing and resource inadequacy as established correlates of missed nursing care [63,64,65,66,67,68,69]. Because staffing, workload, and resource availability were not independently measured or controlled in the present model, the negative IPC–MNC association should not be interpreted as indicating that adaptive coordination is more important than these structural conditions. Instead, the finding adds an interpersonal dimension to the existing literature by showing that, within this sample, nurses’ perceptions of coordination were statistically related to their perceptions of missed nursing care in a remote and culturally diverse hospital context.

This distinction gives the subsection its main interpretive value: IPC was not uniformly related to every antecedent capability, but it was the coordination construct most consistently represented in the significant indirect associations with perceived MNC. That pattern is more specific than a general claim that teamwork is beneficial. It suggests that the relationship between cognitive safety capabilities, professional resilience, and perceived missed care is partly reflected in how nurses report coordination across professional boundaries. The interpretation remains bounded by the cross-sectional, purposive-sampling design and the use of self-reported measures, so the findings describe a pattern of statistical association rather than a causal sequence among the constructs.

### 4.3. Intercultural Communication Competence as a Boundary Condition

The moderation findings add a contextual dimension to the association between Interprofessional Adaptive Coordination (IPC) and perceived Missed Nursing Care (MNC). The significant IPC × Intercultural Communication Competence (ITCC) interaction (β = −0.226, *p* = 0.020) indicates that the IPC–MNC association differed across levels of ITCC. This pattern is consistent with research showing that communication across cultural and linguistic differences is relevant to healthcare interactions, particularly where patient beliefs, expectations, and communication preferences form part of routine care [13,14,15,72,73,74,75,76]. Rather than indicating a general moderating role for ITCC across the model, however, the present finding is specific to the IPC–MNC association. This distinction is important because ITCC was not statistically related to the NRM–MNC association.

The interpretation of this finding also needs to remain aligned with how ITCC was measured. The indicators in the present study focused on nurses’ communication with patients, including recognition of patient beliefs, customs, expectations, and culturally appropriate communication practices [42,43,44]. ITCC therefore represents an individual, patient-facing communication competence, rather than a measure of intercultural communication within the healthcare team or an organizational cultural climate. Within this measurement boundary, the interaction suggests that the statistical association between nurses’ reported IPC and perceived MNC varied according to their patient-facing intercultural communication competence. Previous research similarly emphasizes the role of culturally competent communication in reducing communication barriers and supporting understanding during healthcare encounters [13,14,15,72,73,74,75]. The finding should consequently be interpreted as a contextual association at the individual reporting level, rather than as evidence that ITCC itself produces differences in coordination or missed nursing care.

The simple-slope pattern provides further detail to this interaction. At −1 SD ITCC, the plotted IPC–MNC slope was relatively flat and positive; at the mean level, the slope was slightly negative; and at +1 SD ITCC, the slope was more clearly negative and steeper. The relevant feature is therefore not simply the statistical significance of the interaction term, but the differing direction and steepness of the IPC–MNC association across moderator levels. The positive slope observed at lower ITCC is not readily explained by the available data and should not be interpreted as evidence that greater coordination is associated with more missed care in any causal sense. It is more appropriately treated as an empirical feature of the observed interaction that warrants further investigation. Moreover, because the figure displays conditional slopes rather than providing separate inferential tests for each slope, the direction of a plotted line should not be taken as evidence that the corresponding simple slope is independently statistically significant.

The non-significant ITCC × NRM interaction provides an important contrast. The interaction coefficient was negative (β = −0.184) but did not meet the prespecified significance criterion at α = 0.05 (*p* = 0.053). The estimate was therefore close to, but above, the conventional threshold. This pattern does not provide sufficient statistical evidence for a moderating association between ITCC and the NRM–MNC relationship under the study’s testing criterion. At the same time, the estimate should not be treated as substantively equivalent to a null finding without qualification. The contrast between the two interaction terms is informative because the statistically supported interaction involved IPC, a relational construct concerning coordination across professional roles, whereas the NRM pathway concerned an individual professional capability. The observed pattern is therefore more compatible with a pathway-specific contextual association than with a broad moderating role for ITCC across MNC-related relationships.

This distinction has particular relevance in the South Papua setting. Papua presents a healthcare context in which nurses encounter culturally diverse patients and locally embedded beliefs and practices that can shape communication during care [10,11,76]. Research on nursing in remote Indonesian settings also documents the working conditions associated with geographic isolation [9]. These contextual features make patient-facing intercultural communication a relevant consideration when examining how nurses report their experiences of coordination and missed care. However, the present study did not directly measure intercultural communication among team members, team cultural climate, or institutional cultural practices. Nor did it independently measure staffing, workload, or resource availability. The Papua context should therefore be regarded as an important setting for interpreting the observed pattern, not as a demonstrated explanation for the interaction.

The value of this finding lies in its specificity rather than breadth. ITCC was not associated with variation in every MNC-related pathway; its moderating association was observed for IPC and not for NRM. This pattern is consistent with the possibility that patient-facing intercultural communication competence is more closely related to an association involving coordination and interaction than to one involving an individual resilience capability. Such an interpretation remains tentative because the study used cross-sectional, self-reported data and purposive sampling. The findings therefore describe differences in the statistical association between IPC and perceived MNC across levels of ITCC within the participating nurses, without establishing temporal ordering or causal direction.

### 4.4. Theoretical Contributions

The theoretical contribution of this study lies in providing an explanatory structural account of how cognitive, individual, relational, and contextual capabilities are statistically connected within the patient-safety domain. The findings do not constitute a confirmatory test of Sensemaking Theory; rather, they extend its explanatory application by examining whether different forms of safety-related cognition are associated with adaptive professional and interprofessional capabilities within the same empirical model. The positive associations of risk pattern recognition and safety sensemaking with nurse moral resilience and interprofessional adaptive coordination, together with the distinct pattern for learning from events orientation, indicate that cognitive safety capabilities are not uniformly related across individual and relational domains [30,31,32,33,34,35,36,37,38,39]. This differentiation is theoretically useful because it moves beyond treating “safety capability” as a single, undifferentiated construct and instead suggests that recognition, interpretation, and learning from events occupy distinguishable positions within an explanatory structural framework.

A second contribution concerns the relational positioning of nurse moral resilience. Previous research has primarily examined moral resilience in relation to moral distress, burnout, moral injury, and professional adversity [48,49,50,51,52,60]. In the present model, NRM was positively associated with IPC but not directly with MNC, while a statistically significant indirect association with MNC was observed through IPC [79]. This pattern adds an explanatory dimension to the moral-resilience literature by placing an individual professional capability within a broader relational configuration of care. The finding does not establish a causal mediation mechanism; rather, it suggests that the statistical relationship between NRM and perceived MNC is represented differently when IPC is considered within the structural model. This distinction is theoretically relevant because it separates the individual capability of maintaining professional integrity from the relational process of coordinating care, while examining their interconnection in the same explanatory framework.

A third contribution is the empirical differentiation between immediate safety cognition and event-oriented learning. RPR and SSM were positively associated with IPC, whereas LEO was not significantly associated with IPC despite its positive association with NRM. This pattern is consistent with literature distinguishing event learning and continuous quality improvement from the day-to-day processes of interprofessional coordination [59]. It suggests that the cognitive capabilities most closely associated with recognizing and interpreting current safety situations may have a different structural relationship with coordination than an orientation toward reflecting on previous events. The theoretical value lies not in establishing a hierarchy among these capabilities, but in showing that they are non-equivalent dimensions within the explanatory structure of patient-safety work.

The study further contributes by specifying intercultural communication competence as a pathway-specific contextual condition rather than a universal moderator of patient-safety relationships. The significant interaction between ITCC and IPC in relation to MNC, contrasted with the non-significant ITCC × NRM interaction, indicates that the moderating pattern was specific to the coordination pathway. This is conceptually important because ITCC in this study represents an individual, patient-facing communication competence, rather than team-level intercultural communication or organizational cultural climate [42,43,44]. The finding therefore extends the interpretation of intercultural communication beyond a general communication competency by positioning it as a contextual characteristic associated with variation in the IPC–MNC relationship. This interpretation is particularly relevant to multicultural healthcare contexts, including Papua, where cultural differences and local communication practices form part of the clinical environment [10,11,76].

A further contribution concerns the contextual boundary of the proposed framework. Research from remote Indonesian settings has documented challenges associated with geographically isolated nursing practice [9], while research in Papua has highlighted the relevance of cultural context to healthcare interactions [10,11,76]. The present study does not propose a Papua-specific theory. Instead, it provides an explanatory application of the framework within a remote and culturally diverse hospital setting that is less represented in the patient-safety literature. This contextualization is important because the observed structural associations should be interpreted as evidence from the participating hospitals and nurses, rather than assumed to represent a universal pattern across healthcare systems.

The theoretical value of the model is therefore best understood in terms of configuration and differentiation rather than causal confirmation. The findings place cognitive safety capabilities, moral resilience, interprofessional coordination, and intercultural communication within a single explanatory structure while preserving their conceptual distinctions. The model does not establish temporal ordering, causal effects, or the relative importance of these capabilities compared with staffing, workload, or resource conditions. Instead, it identifies a pattern of statistical relationships that can inform subsequent theory development and empirical testing using longitudinal, multilevel, or probability-based designs. This positioning is consistent with the explanatory use of PLS-SEM in complex latent-variable models and avoids extending cross-sectional associations into causal claims [29].

### 4.5. Practical Implications for Healthcare Organizations and Public Health Practice

The findings have several practical implications for healthcare organizations serving remote and culturally diverse populations. The statistically significant negative association between Interprofessional Adaptive Coordination (IPC) and perceived Missed Nursing Care (MNC) identifies day-to-day coordination as a relevant organizational concern in relation to continuity and completeness of nursing care. Rather than interpreting this finding as evidence that stronger coordination reduces missed care, hospital managers could use it as a basis for reviewing how information is exchanged, responsibilities are clarified, and care activities are coordinated across professional roles. Practical activities such as interdisciplinary safety huddles, structured handovers, multidisciplinary rounds, and case-based safety reviews may provide feasible points within existing workflows for identifying coordination problems and discussing care that may be delayed or left incomplete. These practices should be considered alongside staffing, workload, and resource conditions, which remain established correlates of missed nursing care [48,53,54,55,56,57,62,63,64,65,66,67,68,69].

The differentiated pattern across RPR, SSM, and LEO also has implications for the design of patient-safety activities. The positive associations of RPR and SSM with IPC, together with the absence of a statistically significant LEO–IPC association, suggest that organizations may benefit from distinguishing between recognizing safety signals, making sense of complex situations, and learning from previous events when designing safety routines. Accordingly, structured incident reviews, multidisciplinary debriefings, brief scenario-based exercises, and reflective case discussions could be incorporated into existing clinical activities rather than established as separate programs. In remote hospitals, this approach may be particularly practical where formal training opportunities are limited, and routine clinical encounters provide the main setting for professional learning. These activities should be regarded as organizational practices for further evaluation, not as interventions demonstrated by the present study to change missed nursing care [29,30,35,36,37,59].

The findings concerning NRM and its indirect association with MNC through IPC also suggest a more integrated approach to professional resilience and collaborative practice. NRM was positively associated with IPC, while its direct association with MNC was not statistically significant and its indirect association through IPC was statistically significant. This pattern may justify considering peer reflection, interprofessional case discussion, and team-based problem-solving as complementary components of resilience-oriented organizational practice. Such activities could provide opportunities to consider professional integrity, and coordination demands within the same clinical setting. They should not, however, be presented as evidence-based interventions for reducing missed nursing care, because the present cross-sectional design does not establish intervention effects [48,49,50,51,52,60,61,81].

The significant ITCC × IPC interaction offers a more specific practical consideration for hospitals serving culturally diverse patients. Because ITCC in this study represents individual, patient-facing intercultural communication competence, the practical implication is not to treat it as a proxy for team cultural climate or intercultural competence among professional groups [42,43,44]. Instead, organizations could incorporate culturally responsive communication into existing coordination practices through brief communication guidance for culturally diverse patient encounters, discussion of culturally complex cases during multidisciplinary rounds, and reflective review of communication difficulties identified in routine care. This may be particularly relevant in South Papua, where local cultural context and geographically remote practice form an important part of the healthcare environment [9,10,11,75,76]. The observed moderation should nevertheless be treated as a basis for further organizational evaluation rather than as evidence that intercultural communication training itself will produce better care outcomes.

From a healthcare-quality and public-health perspective, the practical relevance of these findings is therefore best located at the level of frontline care organization. Missed nursing care has been examined in relation to staffing, workload, resource adequacy, patient-safety culture, and care prioritization [63,64,65,66,67,68,69], while remote Indonesian settings present additional operational and contextual considerations [9,10,11]. The present findings add coordination and patient-facing intercultural communication as factors that may be considered alongside these established conditions when organizations examine the continuity and completeness of nursing care. They do not provide evidence of effects on patient outcomes, service utilization, or population health indicators, because these outcomes were not measured. Accordingly, the public-health relevance of the study lies in informing how frontline care processes can be examined and strengthened in context, particularly in remote and culturally diverse hospitals, rather than in claiming downstream population-level effects.

## 5. Limitations and Future Research

Several considerations should be taken into account when interpreting the findings. First, the measurement of Missed Nursing Care (MNC) involved contextual adaptation of indicators drawn from established instruments, including the MINCS, the original MISSCARE Survey, and the revised MISSCARE Survey [6,45,46]. This approach provided content that was relevant to the participating nursing context, but contextual adaptation may also narrow or modify the representation of the broader missed-care construct. Although the questionnaire underwent bilingual forward translation, multidisciplinary expert review, content-validity assessment, and pilot testing, a formal back-translation procedure was not performed. Consequently, residual cross-language equivalence issues cannot be completely excluded. In the present study, one candidate indicator was not retained following the measurement assessment, which may have further reduced the coverage of certain aspects of missed nursing care. Future research could examine the adapted MNC measure in larger and more diverse nursing populations and compare its performance with established frequency-based instruments to assess the stability and breadth of the construct across settings.

Second, the high mean scores observed for the retained MNC indicators suggest that the response distribution may have been concentrated toward the upper end of the scale. This pattern may limit differentiation among respondents reporting different levels of perceived missed care and could partly reflect the agreement-based response format used in the questionnaire. The findings should therefore be interpreted as nurses’ perceptions of missed or incomplete care within the measurement context rather than as direct estimates of the frequency of care omissions. Future studies could examine alternative response formats, include frequency-based MNC measures alongside agreement-based indicators, and use item-level distributional diagnostics to determine whether similar ceiling patterns are observed in other hospital settings.

Third, the interpretation of Intercultural Communication Competence (ITCC) is bounded by the content of its indicators. The items primarily capture nurses’ patient-facing communication competence, including attention to cultural beliefs, customs, expectations, and communication preferences [42,43,44]. Consequently, the construct should not be assumed to represent intercultural communication within professional teams or an organizational cultural climate. This distinction is particularly relevant to the observed moderation of the IPC–MNC association. Future research could distinguish between patient-facing intercultural communication competence and team-level intercultural communication, allowing these dimensions to be examined separately and potentially providing a more precise understanding of their relationships with interprofessional coordination and missed nursing care.

Fourth, the operationalization of Interprofessional Adaptive Coordination (IPC) should also be considered in light of the specific indicators used in this study. Although the construct was conceptualized around communication, role adjustment, and coordination under changing care demands, the indicators reflect nurses’ reported experiences of interprofessional interaction within their clinical context [40,41]. This contextualized measurement provides useful information about nurses’ experiences, but further examination is warranted to determine whether adaptive coordination can be more clearly distinguished from broader interprofessional collaboration or team-climate constructs. Future studies could incorporate indicators that explicitly capture dynamic role adjustment, coordination under uncertainty, and adaptation to changing patient-care demands, together with perspectives from multiple professional groups.

Fifth, the study used a single self-reported measurement source, which may have introduced shared reporting tendencies across constructs. The full-collinearity assessment did not indicate problematic collinearity [78], but this diagnostic should not be interpreted as completely excluding common-source or response-related bias. This consideration is particularly relevant for MNC because perceptions of care omissions may be sensitive to social desirability, recall, and individual interpretation. Future research could combine nurse-reported measures with complementary sources, such as nursing audits, direct observation, incident-reporting data, or selected administrative indicators, to examine whether the observed structural patterns are also evident across different sources of information.

Finally, the South Papua context provides an important setting for the findings but also warrants further comparative examination. The participating hospitals operate within a geographically remote and culturally diverse environment, and previous research has highlighted the distinctive challenges associated with nursing practice and healthcare communication in Papua and other remote Indonesian settings [9,10,11,75,76]. The present findings therefore provide context-specific evidence, while leaving open whether similar structural patterns would be observed under different organizational and cultural conditions. Future research could replicate the model across hospitals with different levels of geographic remoteness, workforce availability, and cultural diversity to examine the consistency of the observed associations and to identify which relationships remain stable across contexts.

## 6. Conclusions

Missed nursing care is not simply a matter of whether individual nurses possess sufficient knowledge, resilience, or commitment. In remote and culturally diverse healthcare environments, it is also embedded in how safety is recognized, interpreted, discussed, and coordinated in everyday clinical work. By bringing these dimensions into a single explanatory framework, this study offers a more relational and context-sensitive way of understanding missed nursing care among frontline nurses in South Papua, Indonesia.

The central insight of the study is the distinctive position of interprofessional adaptive coordination within this configuration. Cognitive safety capabilities and nurse moral resilience were not examined as isolated attributes; their relevance becomes more meaningful when considered alongside the way care is coordinated across professional boundaries. The findings also point to a context-specific role for intercultural communication competence. Its relevance emerged most clearly in the relationship between interprofessional adaptive coordination and perceived missed nursing care, suggesting that its patient-safety significance may depend in part on the interaction and coordination context in which nursing care is organized.

This perspective adds an important dimension to the study of missed nursing care: the phenomenon can be examined not only through what nurses individually possess, but also through how cognitive, professional, relational, and cultural capabilities are positioned together within the care environment. In South Papua, this framing is particularly meaningful because nursing practice takes place at the intersection of geographic remoteness, workforce realities, and cultural diversity. The study therefore offers an explanatory framework that connects individual nursing capabilities with the relational organization of care, while leaving room for established structural conditions such as staffing and resources. Its broader value lies in providing a basis for future research and context-sensitive patient-safety practice in remote and culturally diverse healthcare systems.

## Figures and Tables

**Table 1:** Conceptual Definition.

Constructs	Conceptual Definition	References
Risk Pattern Recognition (RPR)	The capability of nurses to recognize and interpret recurring patterns, salient cues, and potential warning signs that may indicate patient safety risks, drawing on clinical experience, patient information, and contextual knowledge.	[31,32]
Safety Sensemaking (SSM)	The capability of nurses to interpret patient safety concerns, organize relevant information, and develop an understanding of complex or uncertain clinical situations that can inform safety-related decisions and responses.	[30,33,34]
Learning From Events Orientation (LEO	The extent to which nurses are oriented toward learning from patient safety events through reflection, analysis, discussion and dissemination of lessons, and the translation of learning into improvements in clinical practice.	[35,36,37]
Nurse Moral Resilience	The capability of nurses to preserve or restore professional integrity when facing moral adversity, ethical tensions, and competing obligations in clinical practice.	[25,38,39]
Interprofessional Adaptive Coordination (IPC)	The capability of healthcare professionals across disciplines to coordinate flexibly and responsively through information exchange, role adjustment, collaborative decision-making, and coordination of tasks in response to changing patient care demands.	[23,40,41]
Intercultural Communication Competence (ITCC)	The extent to which individual nurses adapt their communication with patients to cultural differences, including differences in values, beliefs, health literacy, expectations, and communication preferences, using culturally sensitive and effective communication strategies.	[15,42,44]
Missed Nursing Care (MNC)	The perceived occurrence of delayed, incomplete, or omitted nursing care activities, including aspects of basic needs, patient safety, emotional support, privacy, and patient education within routine nursing practice	[3,4,6,45,46]

**Table 2:** Participant Characteristic (*n* = 312).

Characteristics	Category	*n*	%
Sex	Male	94	30.1
	Female	218	69.9
Age	20–30 years	124	39.7
	31–40 years	132	42.3
	41–50 years	48	15.4
	>50 years	8	2.6
Education Level	Diploma (D3)	110	35.3
	Bachelor’s Degree (S1)	91	29.2
	Nurse Profession (Ners)	92	29.5
	Master’s Degree or Higher	19	6.1
Working Experience	<1 year	15	4.8
	1–5 years	129	41.3
	6–10 years	89	28.5
	>10 years	79	25.3
Employment Status	Civil Servant	186	59.6
	Non-Civil Servant	126	40.4
Ethnicity	Non-Indigenous Papuan	116	37.2
	Indigenous Papuan	196	62.8
Work Unit	Inpatient Ward	138	44.2
	ICU	38	12.2
	Operating Room	37	11.9
	Hemodialysis Unit	23	7.4
	Emergency Department (ED)	73	23.4
	Others	3	1

**Table 4:** Heterotrait–monotrait ratio (HTMT).

Variables	ITCC	IPC	LEO	MNC	NRM	RPR	SSM	ITCC × NRM	ITCC × IPC
ITCC									
IPC	0.892								
LEO	0.642	0.568							
MNC	0.110	0.194	0.046						
NRM	0.766	0.822	0.603	0.129					
RPR	0.834	0.790	0.506	0.145	0.778				
SSM	0.768	0.842	0.666	0.078	0.867	0.770			
ITCC × NRM	0.153	0.201	0.253	0.573	0.233	0.121	0.164		
ITCC × IPC	0.082	0.083	0.211	0.577	0.175	0.085	0.173	0.828	

ITCC = intercultural communication, IPC = interprofessional adaptive coordination, LEO = learning from even orientation, MNC = missed nursing care, NRM = nurse’s moral resilience, RPR = risk pattern recognition, SSM = safety sensemaking.

**Table 5:** Cross-Validated Predictive Ability Test (CVPAT).

Variable/Model	PLS-SEM vs. Indicator Average (IA)				PLS-SEM vs. Linear Model (LM)			
	PLS Loss	IA Loss	Average Loss Difference	*p*-Value	PLS Loss	LM Loss	Average Loss Difference	*p*-Value
Interprofessional Adaptive Coordination	0.264	0.394	−0.130	0.000	0.264	0.290	−0.026	0.000
Nurse Moral Resilience	0.190	0.292	−0.102	0.000	0.190	0.159	0.031	0.044
Missed Nursing Care	0.309	0.392	−0.083	0.000	0.309	0.419	−0.110	0.000
Overall (Model)	0.259	0.366	−0.107	0.000	0.259	0.298	−0.039	0.000

**Table 6:** Hypothesis Testing Result.

	Hypotheses	Coefficient (β)	*p*-Values	Confidence Interval		Decision
				Lower 5%	Upper 95%	
H1	Risk Pattern Recognition → Nurse Moral Resilience	0.361	<0.001	0.279	0.444	Hypothesis Supported
H2	Risk Pattern Recognition → Interprofessional Adaptive Coordination	0.294	<0.001	0.190	0.405	Hypothesis Supported
H3	Safety Sensemaking → Nurse Moral Resilience	0.414	<0.001	0.328	0.494	Hypothesis Supported
H4	Safety Sensemaking → Interprofessional Adaptive Coordination	0.241	<0.001	0.128	0.350	Hypothesis Supported
H5	Learning From Events Orientation → Nurse Moral Resilience	0.135	0.001	0.065	0.214	Hypothesis Supported
H6	Learning From Events Orientation → Interprofessional Adaptive Coordination	0.065	0.107	−0.017	0.156	Hypothesis Not Supported
H7	Nurse Moral Resilience → Missed Nursing Care	0.133	0.104	−0.046	0.296	Hypothesis Not Supported
H8	Interprofessional Adaptive Coordination → Missed Nursing Care	−0.220	0.027	−0.367	−0.006	Hypothesis Supported
H9	Intercultural Communication Competence moderates Nurse Moral Resilience → Missed Nursing Care	−0.184	0.053	−0.325	0.019	Hypothesis Not Supported
H10	Intercultural Communication Competence moderates Interprofessional Adaptive Coordination → Missed Nursing Care	−0.226	0.020	−0.369	−0.044	Hypothesis Supported
H11	Nurse Moral Resilience → Interprofessional Adaptive Coordination	0.296	<0.001	0.178	0.412	Hypothesis Supported

**Table 7:** Mediation Analysis Results.

Specific Indirect Path	β	*p*-Value	Bootstrap Interval (5–95%)	Interpretation
LEO → NRM → MNC	0.018	0.140	[−0.006, 0.047]	Not statistically significant
LEO → IPC → MNC	−0.014	0.136	[−0.035, 0.006]	Not statistically significant
RPR → NRM → MNC	0.048	0.108	[−0.018, 0.108]	Not statistically significant
RPR → IPC → MNC	−0.065	0.043	[−0.122, 0.002]	Statistically significant
SSM → NRM → MNC	0.055	0.110	[−0.019, 0.126]	Not statistically significant
SSM → IPC → MNC	−0.053	0.040	[−0.096, 0.001]	Statistically significant
NRM → IPC → MNC	−0.065	0.046	[−0.124, 0.002]	Statistically significant

β = standardized specific indirect effect. Bootstrap intervals are reported using the 5th and 95th percentile bounds. LEO = Learning from Events Orientation; RPR = Risk Pattern Recognition; SSM = Safety Sensemaking. NRM = Nurse Moral Resilience; IPC = Interprofessional Adaptive Coordination; MNC = Missed Nursing Care.

**Table 8:** Conditional Effect at ITCC Levels.

ITCC Level	Conditional β	Sample Mean	STDEV (SE)	*p*-Value	Bootstrap Interval (5–95%)	Interpretation
−1 SD (Low ITCC)	0.006	0.020	0.152	0.484	[−0.218, 0.271]	Positive but not statistically significant association
Mean ITCC	−0.220	−0.194	0.115	0.027	[−0.367, 0.006]	Negative and statistically significant association
+1 SD (High ITCC)	−0.447	−0.408	0.167	0.004	[−0.639, −0.136]	Stronger negative and statistically significant association

ITCC = Intercultural Communication Competence; β = standardized conditional association; STDEV = standard deviation of the bootstrap distribution; *p* < 0.05 indicates statistical significance.

## Data Availability

The datasets generated and/or analyzed during the current study are not publicly available due to ethical and confidentiality considerations involving healthcare professionals and institutional data from four hospitals in Merauke Regency.

## Supplementary Materials

The following supporting information can be downloaded at: https://www.mdpi.com/article/10.3390/healthcare14203470/s1, Table S1: Response Distribution and Descriptive Statistics of Retained Missed Nursing Care Indicators; Table S2: Sensitivity Analysis of the Principal IPC–MNC Association and ITCC × IPC Interaction After Excluding MNC6.
