# Supplementary material for: Associations of Interprofessional Adaptive Coordination and Intercultural Communication Competence with Missed Nursing Care in Remote Hospitals in South Papua, Indonesia
Source: Healthcare (Basel). 2026 Oct 15;14(20):3470. doi: 10.3390/healthcare14203470 (PMC13704080; doi:10.3390/healthcare14203470)
Supplement: Supplementary file 1 [file healthcare-14-03470-s001.zip › healthcare-4574405-supplementary.pdf]

Supplementary Table S1. Response Distribution and Descriptive Statistics of Retained Missed Nursing Care Indicators.

Indicator	Mean	SD	Observed Range	Response 2, n (%)	Response 3, n (%)	Response 4, n (%)	Response 5, n (%)
MNC1	4.556	0.614	3–5	0 (0.0%)	18 (6.5%)	87 (31.4%)	172 (62.1%)
MNC2	4.635	0.601	3–5	0 (0.0%)	18 (6.5%)	65 (23.5%)	194 (70.0%)
MNC3	4.596	0.615	3–5	0 (0.0%)	19 (6.9%)	74 (26.7%)	184 (66.4%)
MNC4	4.606	0.589	3–5	0 (0.0%)	15 (5.4%)	79 (28.5%)	183 (66.1%)
MNC6	4.527	0.693	2–5	1 (0.4%)	29 (10.5%)	70 (25.3%)	177 (63.9%)

Note. MNC = missed nursing care; SD = standard deviation. Items were rated on a five-point agreement scale from 1 (*strongly disagree*) to 5 (*strongly agree*). No responses in category 1 were observed. Higher scores indicate stronger agreement that the described missed-care condition occurred.

Supplementary Table S2. Sensitivity Analysis of the Principal IPC–MNC Association and ITCC × IPC Interaction After Excluding MNC6.

Structural Path	Original Model (MNC6 Included) β	STDEV	p-value	Sensitivity Model (MNC6 Excluded) β	STDEV	p-value	Δβ	Stability
IPC → MNC	−0.220	0.115	.027	−0.193	0.111	.041	+0.027	Direction unchanged; slightly attenuated; statistical conclusion unchanged
ITCC × IPC → MNC	−0.226	0.111	.020	−0.239	0.115	.019	−0.013	Direction and magnitude stable; statistical conclusion unchanged

Note. IPC = Interprofessional Adaptive Coordination; ITCC = Intercultural Communication Competence; MNC = Missed Nursing Care; β = standardized path coefficient; STDEV = standard deviation of the bootstrap distribution; Δβ = β (MNC6 excluded) − β (MNC6 included). The sensitivity model was re-estimated after removing MNC6 while retaining the structural specification of the original model.
